# A SOX17-PDGFB signaling axis regulates aortic root development

**DOI:** 10.1038/s41467-022-31815-1

**Published:** 2022-07-13

**Authors:** Pengfei Lu, Ping Wang, Bingruo Wu, Yidong Wang, Yang Liu, Wei Cheng, Xuhui Feng, Xinchun Yuan, Miriam M. Atteya, Haleigh Ferro, Yukiko Sugi, Grant Rydquist, Mahdi Esmaily, Jonathan T. Butcher, Ching-Pin Chang, Jack Lenz, Deyou Zheng, Bin Zhou

**Affiliations:** 1 Department of Genetics, Albert Einstein College of Medicine, Bronx, NY USA; 2 School of Medical Imaging, Tianjin Medical University, Tianjin, China; 3 Cardiovascular Research Center, School of Basic Medical Sciences, Jiaotong University, Xi’an, Shanxi China; 4 The First Affiliated Hospital of Nanchang University, Nanchang, Jiangxi China; 5 Department of Regenerative Medicine and Cell Biology, Medical University of South Carolina, Charleston, SC USA; 6 School of Mechanical and Aerospace Engineering, Cornell University, Ithaca, NY USA; 7 School of Biomedical Engineering, Cornell University, Ithaca, NY USA; 8 Departments of Microbiology & Immunology, Albert Einstein College of Medicine, Bronx, NY USA; 9 Departments of Neurology and Neuroscience, Albert Einstein College of Medicine, Bronx, NY USA; 10 Departments of Pediatrics and Medicine (Cardiology), Albert Einstein College of Medicine, Bronx, NY USA

**Keywords:** Disease model, Cell proliferation, Heart development

## Abstract

Developmental etiologies causing complex congenital aortic root abnormalities are unknown. Here we show that deletion of *Sox17* in aortic root endothelium in mice causes underdeveloped aortic root leading to a bicuspid aortic valve due to the absence of non-coronary leaflet and mispositioned left coronary ostium. The respective defects are associated with reduced proliferation of non-coronary leaflet mesenchyme and aortic root smooth muscle derived from the second heart field cardiomyocytes. Mechanistically, SOX17 occupies a *Pdgfb* transcriptional enhancer to promote its transcription and *Sox17* deletion inhibits the endothelial *Pdgfb* transcription and PDGFB growth signaling to the non-coronary leaflet mesenchyme. Restoration of PDGFB in aortic root endothelium rescues the non-coronary leaflet and left coronary ostium defects in *Sox17* nulls. These data support a SOX17-PDGFB axis underlying aortic root development that is critical for aortic valve and coronary ostium patterning, thereby informing a potential shared disease mechanism for concurrent anomalous aortic valve and coronary arteries.

## Abstract (web summary)

Little is known about the developmental causes of aortic root defects. Here the authors show that the inactivation of Sox17 in aortic root endothelium results in aortic root defects affecting aortic valve and coronary ostium.

## Introduction

The aortic valve and coronary ostia are located within the aortic root, a central cardiovascular structure connecting the aorta to the left ventricle. The aortic valve directs the systemic circulation from the left ventricle to the aorta, whereas the coronary ostia shunt blood from the aorta to the main coronary arteries. Anomalous aortic valve and coronary arteries are significant health problems. For instance, bicuspid aortic valve (BAV) is the most common congenital heart defect, affecting 1–2% of the general population, half of whom develop aortic valve stenosis in late life^1^, whereas malformations of the coronary ostia affect ~1% of the general population and contribute to ~25% of all cases of coronary artery anomalies (CAAs), which can lead to myocardial ischemia, heart failure and sudden cardiac death^2^.

Association of BAV with anomalous coronary arteries has been reported^3,4^. Several studies have uncovered significantly increased prevalence of CAAs, such as the absence of the left coronary artery (LCA) in BAV patients compared with their tricuspid counterparts, and BAV patients with coronary anomalies have more postoperative ischemic cardiac events and increased morbidity^5–9^. Furthermore, recent genome-wide analysis has identified variant loci in patients with aortic valve stenosis associating with BAV and coronary artery disease, suggesting shared genetic predisposition^10^. The intimate anatomic locations of the aortic valve and coronary ostia in a hemodynamic-regulated environment support a shared developmentally-regulated morphogenic mechanism involved in both congenital anomalies^11^. Furthermore, as BAV is inheritable and highly associated with other aorta root abnormalities^12–14^, a shared genetic cause likely underlies these developmental defects^15,16^. Animal and pluripotent stem cell studies that model human genetic mutations, such as variants in *ROBO4*^17^ and GATA4/5/6^18–22^, also support this notion. However, shared genetic and developmental etiologies contributing to BAV and anomalous coronary arteries remain to be defined in animal models.

Among the BAV-associated genetic variants, *NOTCH1* mutations are the first identified to cause familial BAV in humans^12^. In those patients with deficient NOTCH1 signaling, BAV is also accompanied by dilated aortic root and aortic aneurysm^23,24^. In mice, NOTCH1 is predominately expressed in cardiac endocardium and vascular endothelium during development and plays critical roles in the formation of endocardial cushions^25^, cardiac chambers^26^ and vasculogenesis^27^. We have previously shown that inactivation of *Notch1* in the aortic valve endothelium in mice causes BAV, dilated aortic root and CAAs^28^. The *Notch1* deletion inhibits the TNFα signaling that promotes apoptosis necessary for forming a tricuspid aortic valve^28^ and reduces the *Hbegf* expression that counteract the pro-proliferation BMP signaling essential for proper aortic valve growth and maturation^29^.

NOTCH1 signaling is regulated by transcription factor SOX17 (SRY-box 17) for arterial specification and maintenance in zebrafish and mice^30–33^. This study addresses the potential role of SOX17 in aortic root formation using mouse models by targeted deletion of *Sox17* in aortic root endothelium. The deletion causes BAV due to the absence of non-coronary leaflet (NCL) and misplaced left coronary ostium (LCO). By charactering the cellular and molecular changes in the *Sox17* nulls, we show that SOX17 promotes the endothelial *Pdgfb* transcription and PDGFB signaling required for the proliferation of NCL mesenchyme and aortic root smooth muscle, which share a common embryonic origin in the second heart field (SHF)-derived cardiomyocytes. Our study thus uncovers a potential disease mechanism involved in a SOX17-PDGFB regulatory axis for concurrent anomalous aortic valve and coronary arteries.

## Results

### Deletion of *Sox17* in aortic root endothelium

**Fig. 1:**
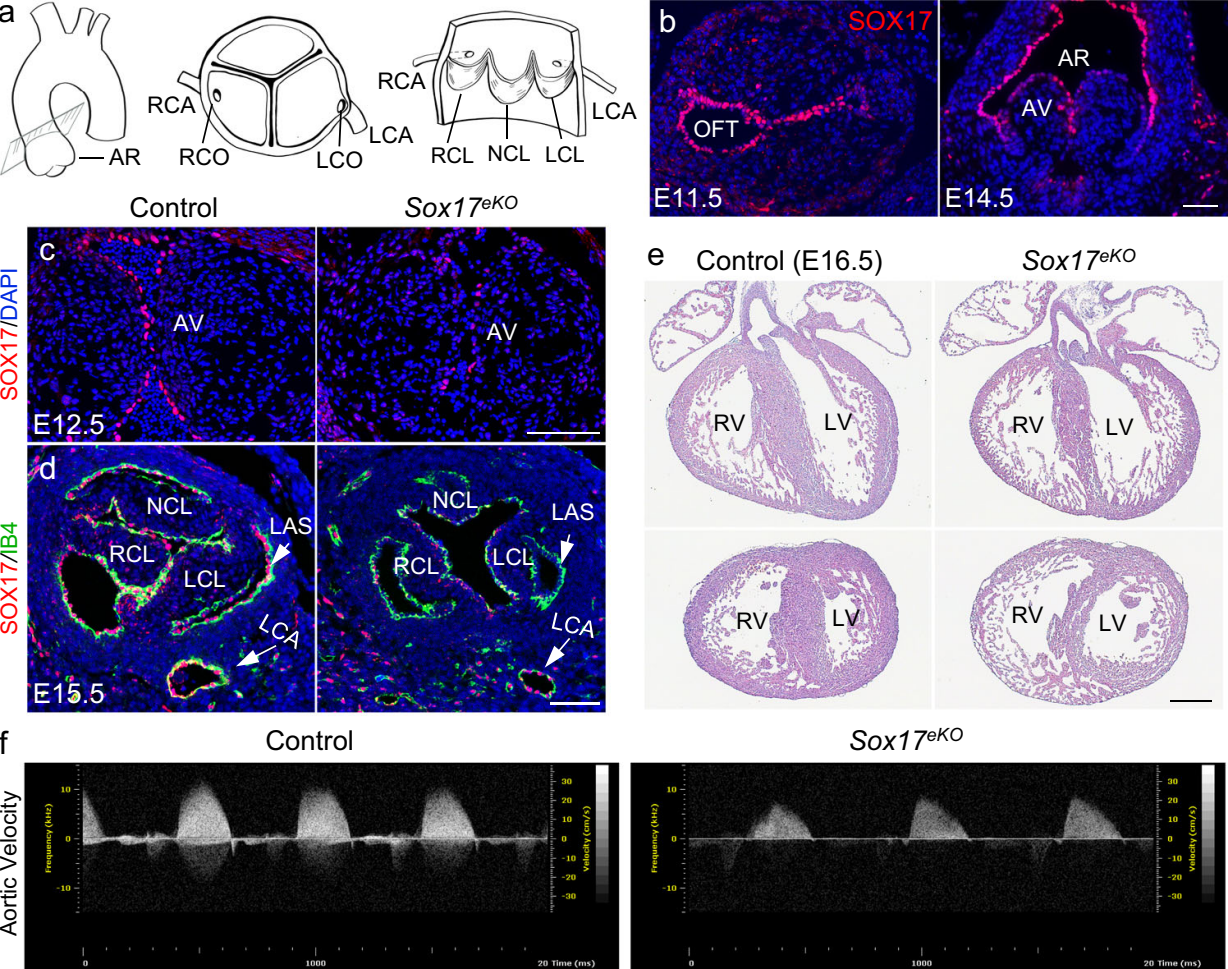
Sox17 expression in the aortic root endothelium is necessary for heart development and embryonic survival. **a** Schematic view of aortic root (AR) shows the anatomic relationship of aortic valve, aortic sinus and coronary ostium. LCL/RCL/NCL: left, right and non-coronary leaflet; LCO/RCO: left and right coronary ostium, LCA/RCA: left and right coronary artery. **b** Representative immunofluorescence (IF) (*n* = 3/group) shows specific and developmentally regulated SOX17 expression (red) in the aortic root endothelium of E11.5 and E14.5 hearts. **c** IF (*n* = 5/group) shows diminished SOX17 expression (red) in the aortic root endothelium of E12.5 *Sox17*^*eKO*^ hearts. AV: aortic valve. **d** IF (*n* = 5/group) shows reduced SOX17 expression (red) in the aortic root endothelium in E15.5 *Sox17*^*eKO*^ hearts. Note that the SOX17 expression in the coronary artery endothelium is not affected by the valve specific deletion using the transgenic Nfatc1-enhancer Cre which expresses specifically in the aortic root endothelium including the valve endothelium. **e** H&E staining (*n* = 5/group) of frontal (top panels) and transverse sections (bottom panels) show thin ventricular wall in all 8 E16.5 *Sox17*^*eKO*^ embryos examined. LV/RV: left and right ventricle. **f** Echocardiography (*n* = 3/group) shows decreased aortic velocity in E16.5 *Sox17*^*eKO*^ hearts. Scale bar: 100 µm in **b**–**d**, 200 µm in **e**.

The aortic root consists of a tricuspid aortic valve with the left, right and non-coronary leaflet (LCL, RCL, NCL), three respective (left, right, non-coronary) aortic sinuses (LAS, RAS, NCS), and two (left and right) coronary ostia (LCO, RCO) (Fig. 1a). Using immunostaining we found that SOX17 is highly expressed in the endothelium of the cardiac outflow tract (OFT) at embryonic day (E) 11.5 when aortic valve leaflets begin to form by remodeling of OFT mesenchyme, and the OFT endothelial expression persists till E14.5 when aortic valve leaflets and respective sinuses are recognizable (Fig. 1b). To address the role of *Sox17* in aortic root formation, we deleted *Sox17* in the OFT endothelium by crossing the floxed *Sox17* (*Sox17*^*f/f*^)^34^ with the transgenic *Nfatc1*-enhancer Cre (*Nfatc1*^*enCre*^) mice as *Nfatc1*^*enCre*^ only starts to express in the OFT around E11.5 when endocardial to mesenchymal transformation (EndMT) is ended and is not expressed in the coronary vascular endothelium^35^. The SOX17 expression in the forming aortic valve endothelium of the E12.5 knockout embryos (*Sox17*^*eKO*^ hereafter) was substantially reduced when compared to their *Sox17*^*f/+*^ or *Sox17*^*f/f*^ littermates (designated as control) (Fig. 1c). The SOX17 inactivation persisted in the aortic root endothelium of the *Sox17*^*eKO*^ heart by E15.5 whereas the SOX17 expression in the coronary artery endothelium and ventricular endocardium was not affected (Fig. 1d and Supplementary Fig. 1, respectively). These observations confirm the tissue specificity and efficacy of the SOX17 inactivation using *Nfatc1*^*enCre*^ that may reveal the SOX17 role in aortic root formation.

### *Sox17* expression in aortic root endothelium is required for heart development

The *Sox17* deletion resulted in the death of *Sox17*^*eKO*^ embryos before birth as all *Sox17*^*eKO*^ embryos were resorbed at E18.5 (Supplementary Table 1). The increased penetrance of lethality from E16.5 to E18.5 likely reflected a time required for developing lethal structural and functional abnormalities. We chose to analyze the cardiac phenotypes at E16.5 when ~21% of *Sox17*^*eKO*^ embryos were found dead at dissection and the survived mutants were comparable to the control littermates in body or heart size. We found by H&E staining that *Sox17*^*eKO*^ embryos developed hypoplastic ventricles (Fig. 1e) associated with decreased aortic velocity (Fig. 1f). Further TUNEL assays revealed significantly increased apoptotic ventricular cardiomyocytes in the *Sox17*^*eKO*^ heart (Supplementary Fig. 2a, b) associated with reduced thickness of the left ventricular free wall (Supplementary Fig. 2c). These observations suggest that aortic valve deficiency and/or reduced left ventricular functions might cause the death of *Sox17*^*eKO*^ embryos.

### *Sox17* deletion in aortic root endothelium causes BAV

**Fig. 2:**
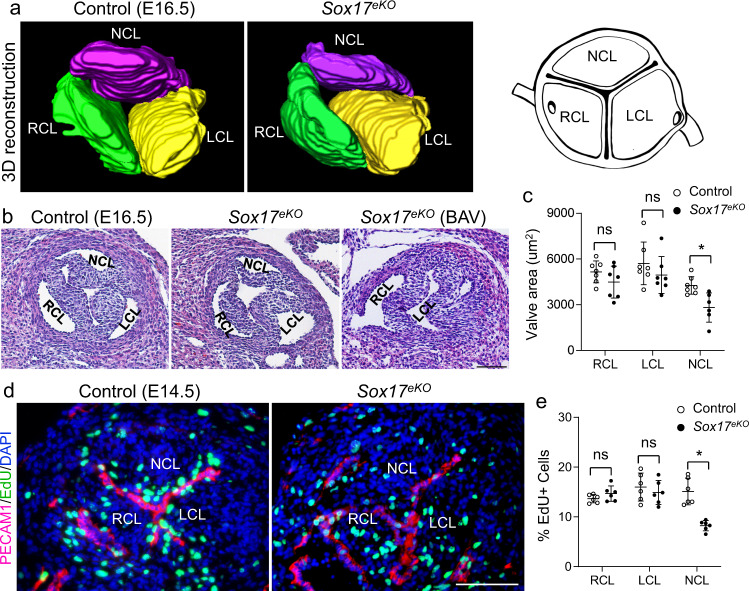
Deletion of Sox17 in the aortic root endothelium causes aortic valve defects. **a** 3D reconstruction of continuous H&E stained sections of aortic root shows reduced NCL volume in the E16.5 *Sox17*^*eKO*^ heart (*n* = 5/group). A schematic view of aortic valve leaflets is on the right. **b** Representative H&E stained transverse sections show underdeveloped NCL and BAV without NCL in the E16.5 *Sox17*^*eKO*^ heart. **c** Quantitative analysis shows significantly decreased area of NCL in E16.5 *Sox17*^*eKO*^ hearts. (*n* = 7 for control, *n* = 6 for *Sox17*^*eKO*^, mean ± SD, unpaired two-tailed *t*-test, *p* = 0.0063 for NCL, *p < 0.05). BAV was excluded from the quantitative analysis. **d** Representative images from EdU (green) assays on sections of E14.5 hearts show reduced cell proliferation in NCL. **e** Quantitative analysis of EdU assays. (*n* = 6/group, mean ± SD, unpaired two-tailed *t*-test, *p* = 0.0002 for NCL, *p < 0.05). Source data are provided as a Source Data file. Scale bars: 100 µm.

We next examined the aortic valve by 3D reconstructions of H&E stained E16.5 heart sections and found underdeveloped NCL in *Sox17*^*eKO*^ hearts (Fig. 2a). Close inspection of individual sections confirmed the undersized NCL and identified BAV without NCL in 4 of 15 (~27%) *Sox17*^*eKO*^ hearts (Fig. 2b). The undersized or absence of NCL reflect the same defect of varying severities likely due to a variable deletion efficiency. Quantification of the area of LCL, RCL or NCL indicated that underdeveloped NCL was a specific phenotype (Fig. 2c). Note that the quantification did not include the BAV cases. Furthermore, quantification of the size of the pulmonary, mitral and tricuspid valve (PV, MV, TV) indicated that their development was not affected by the *Sox17* deletion (Supplementary Fig. 3).

It is known that NCL has the least contribution from EndMT^36,37^. Importantly, the *Nfatc1*^*enCre*^ only starts to express the Cre recombinase around E11.5 when the EndMT process is mostly completed at OFT and atrioventricular canal (AVC). To explain the NCL specific defect, we examined the proliferation and apoptosis of aortic valve cells of E14.5 hearts as aortic valve leaflets grow actively. EdU labeling showed significantly reduced proliferation in NCL, but not LCL and RCL, in the *Sox17*^*eKO*^ hearts (Fig. 2d, e). In contrast, no changes in the EdU labeling were found in PV, MV and TV after the *Sox17* deletion (Supplementary Fig. 4). Furthermore, TUNEL assays did not show significant changes in apoptosis in all four heart valves of the *Sox17*^*eKO*^ heart (Supplementary Fig. 5). These observations demonstrate an essential role for *Sox17* in promoting the NCL growth in our mouse model of BAV due to the absence of NCL.

### *Sox17* deletion in aortic root endothelium impairs aortic root wall development

**Fig. 3:**
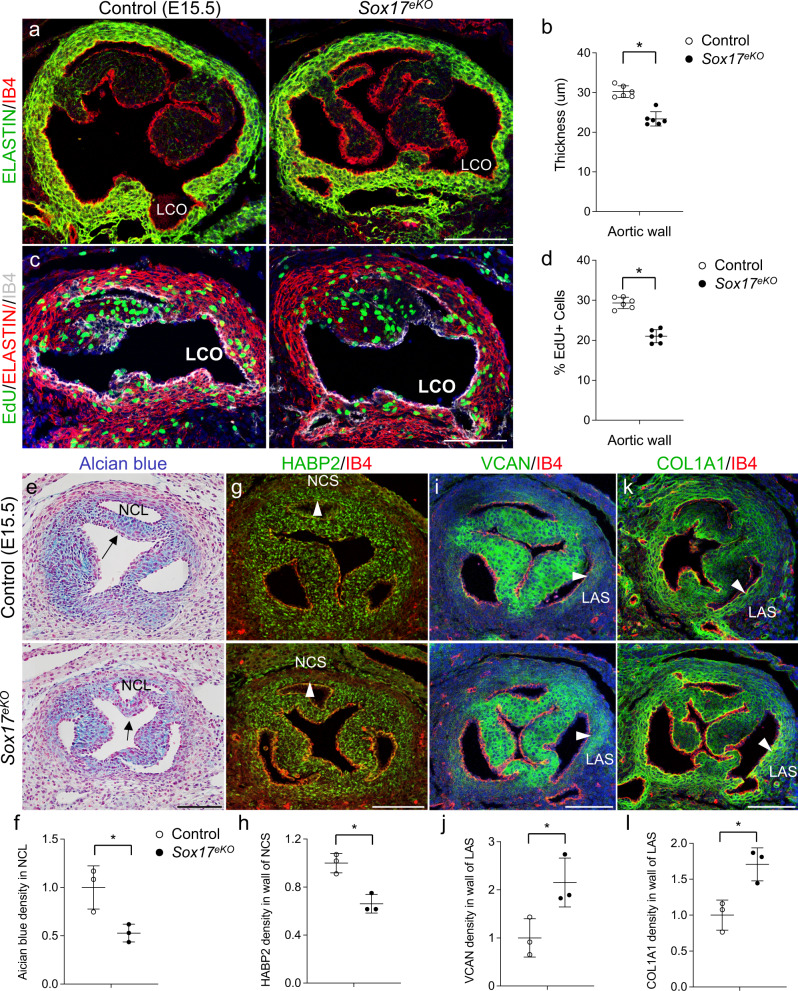
Sox17 deletion in the aortic root endothelium impairs aortic root growth and maturation. **a** IF for ELASTIN (green) shows thin aortic root wall in E15.5 *Sox17*^*eKO*^ hearts. **b** Quantitative analysis shows a significant reduction in the wall thickness. **c** EdU (green) assay shows reduced proliferation of VSMCs in the aortic root wall marked by red ELSTAIN staining in *Sox17*^*eKO*^ hearts. IB4 (white) stains the endothelium. **d** Quantitative analysis shows a significant reduction in VSMC proliferation. (For (**a**–**d**) Figures, *n* = 6/group, mean ± SD, unpaired two-tailed *t*-test, *p* = 0.00003 for (**b**), *p* = 0.000003 for (**d**), *p < 0.05). **e**, **f** Representative Alcian blue staining images and quantitative analysis of E15.5 control and *Sox17*^*eKO*^ hearts show reduced proteoglycans in NCL (arrow) of the *Sox17*^*eKO*^ hearts. **g**, **h** Representative IF images and quantitative analysis of E15.5 control and *Sox17*^*eKO*^ hearts indicate decreased HABP2 (green) in the wall of NCS (arrowhead). **i**, **j** Representative IF images and quantitative analysis of E15.5 control and *Sox17*^*eKO*^ hearts show increased VCAN (green) in the wall of LAS (arrowhead). **k**, **l** Representative IF images and quantitative analysis of E15.5 control and *Sox17*^*eKO*^ hearts showing upregulated COL1A1 (green) in the wall of LAS (arrowhead). In **e**–**l**, *n* = 3/group, mean ± SD, unpaired two-tailed *t*-test, *p* = 0.027 for (**f**), *p* = 0.006 for (**h**), *p* = 0.036 for (**j**), *p* = 0.017 for (**l**), *p < 0.05. Source data are provided as a Source Data file. Scale bars: 100 µm.

To determine whether the *Sox17* deletion in aortic root endothelium might affect aortic root wall development, we conducted immunostaining for ELASTIN, a major extracellular matrix (ECM) component produced by the vascular smooth muscle cells (VSMCs). The results revealed significantly reduced wall thickness of the aortic root in E15.5 *Sox17*^*eKO*^ hearts when compared to control littermates (Fig. 3a, b). Moreover, the thin wall was associated with significantly decreased proliferation of VSMCs (Fig. 3c, d). In contrast, TUNEL assays showed no changes in apoptosis in the aortic root wall of *Sox17*^*eKO*^ hearts (Supplementary Fig. 6). Alcian blue staining (pH 2.5) and immunostaining for HABP2 (Hyaluronic Acid Binding Protein 2) revealed a moderate decreased mesenchymal acid mucins in NCL (Fig. 3e, f, arrow) and HABP2 in the NCS wall (Fig. 3g, h, arrowhead), respectively. In addition, the level of proteoglycan VCAN (VERSICAN) and connective tissue collagen 1A1 (COL1A1) were upregulated in the LAS wall (Fig. 3i–l, arrowhead). These findings support that SOX17 expressed by aortic root endothelium is required for promoting the proliferation of VSMCs and the maturation of aortic root wall.

It is known that LCL and RCL begin to form around E10.5 by cells derived from a combination of the local EndMT at OFT^35,38,39^ and the invasion of the cardiac neural crest cells (NCCs)^40–43^, whereas NCL develops 1 day later from the mesenchymal cells that are largely of the SHF origin^36,37^ that share the gene expression characteristics with the cardiomyocytes^44,45^. The SHF progenitor cells also give rise to VSMCs to form the smooth muscle media of aortic sinuses (i.e., aortic root wall)^46–49^. To determine whether the spatiotemporally dysregulated cell proliferation and ECM maturation resulted from the *Sox17* deletion is related to the shared SHF origin of the NCL mesenchyme and aortic wall muscle, we carried out lineage tracing analysis using the transgenic TnT-Cre (*TnT*^*Cre*^) mouse^50^ and the *R26R*^*fsGFP*^ reporter mouse^51^. The results showed while the TnT expression was not present in the NCL mesenchyme and aortic wall muscle of E13.5 *TnT*^*Cre*^*:R26R*^*fsGFP*^ hearts (Supplementary Fig. 7a), the cardiomyocyte lineage marker GFP was clearly expressed in these areas (Supplementary Fig. 7b). Note that we did not use a SHF progenitor Cre mouse line (*Mef2*^*Cre*^ or *Isl1*^*Cre*^) as it would yield confounding lineage tracing results due to labeling the OFT endothelial cells that undergo EMT becoming mesenchymal cells, in addition to the cardiomyocytes. Instead, we performed immunostaining for ISL1, a SHF progenitor marker^44^. The results showed that ISL1 is preferably expressed in NCL but not LCL and RCL (Supplementary Fig. 7c) and the number of ISL1-expressing cells was significantly reduced in the NCL mesenchyme of the E14.5 *Sox17*^*eKO*^ hearts while the ISL1 expression in the cardiomyocytes was not affected (Supplementary Fig. 7d, e). Thus, both lineage tracing and ISL1 expression data support that the *Sox17* deletion after E11.5 specifically interfere with the proliferation and maturation of the SFH-derived NCL mesenchyme and aortic root muscle. These observations are consistent with the previous findings that the SHF-derived cells populate NCL^44^.

### *Sox17* deletion in aortic root endothelium results in misplaced left coronary ostium

**Fig. 4:**
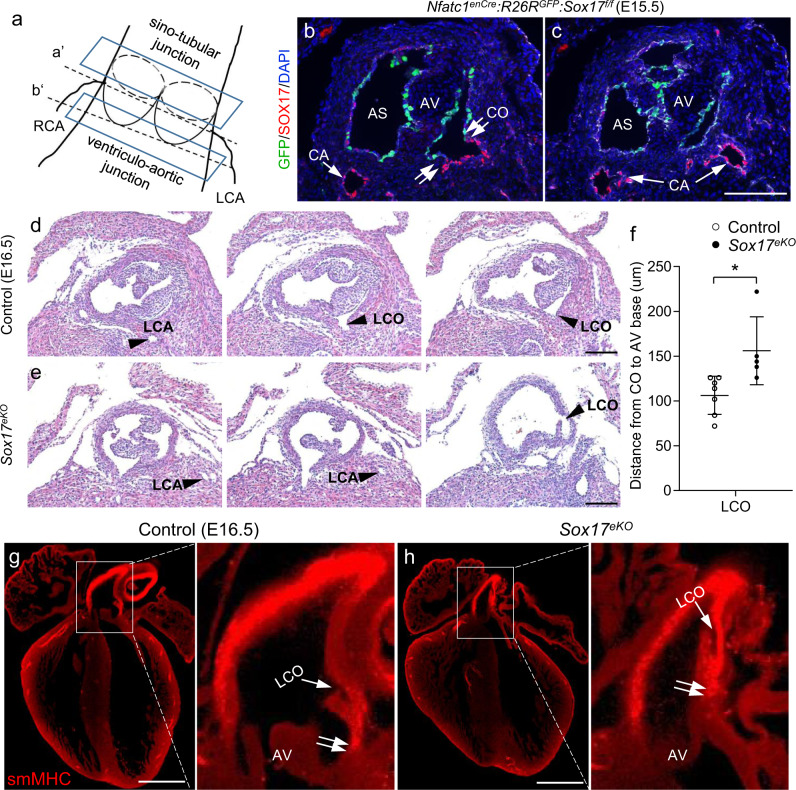
Deletion of Sox17 results in high-takeoff left coronary ostium. **a** Schematic view shows the coronary ostium position within the aortic root. Top plane indicates the sino-tubular junction between the aortic sinus and the ascending aorta. Bottom plane indicates the ventricular-aortic junction between the left ventricle and the aortic root. Dashed line a’ indicates the coronary ostia (origin of each main coronary stem in the respective aortic sinus). Dashed line b’ indicates the base of aortic valve leaflets. **b**, **c** IF images (*n* = 5/group) for the GFP reporter (green, indicating the lineage of *Nfatc1*^*enCre*^*-*marked aortic root endothelial cells) and for SOX17 staining (red) for aortic root and coronary arterial endothelium. Note a sharp tissue boundary (**b**, double arrows) between the GFP-positive/SOX17-deleted aortic root endothelium and the GFP-negative/SOX17-expressing coronary artery endothelium, which marks the coronary entries or ostia. The main coronary arteries are negative for GFP but positive for SOX17, indicating that the deletion does not affect the coronary artery endothelium. **d**, **e** Representative H&E stained continuous sections E16.5 control and *Sox17*^*eKO*^ hearts from the base of aortic valve leaflets to the coronary ostia shows that LCO in E16.5 *Sox17*^*eKO*^ hearts is shifted up and posteriorly toward the left coronary sinus. Arrowheads indicate the main coronary arteries and ostia. **f** Quantitative analysis of (**d**, **e**) confirms the high-takeoff LCO. (*n* = 7 for control, *n* = 5 for *Sox17*^*eKO*^, mean ± SD, unpaired two-tailed *t*-test, *p* = 0.015, *p < 0.05). **g**, **h** Representative smMHC IF images (*n* = 5/group) show the high-takeoff LCO (single arrow) in E16.5 *Sox17*^*eKO*^ hearts and upshifted ventricular (smMHC-negative)-aortic (smMHC-positive) junction (double arrows). Source data are provided as a Source Data file. Scale bars: 100 µm.

As the *Sox17* deletion resulted in hypoplastic heart, we sought to determine whether SOX17 is required for forming the coronary ostium (Fig. 4a). By immunostaining for SOX17 and the lineage marker GFP for aortic root endothelial cells in E15.5 *Nfatc1*^*enCre*^*:R26R*^*GFP*^*:Sox17*^*f/f*^ hearts, we showed that the deletion was restricted to aortic root endothelium as the SOX17 expression in the coronary artery endothelium was not affected (Fig. 4b, c). Specifically, by demonstrating the GFP-positive and the SOX17-negative aortic sinuses as well as the GFP-negative and the SOX17-positive main coronary entries, we could virtually identify the coronary ostium (Fig. 4b, double arrows) and validated that the tissue deletion domain was restricted to aortic root endothelium, which did not affect coronary artery endothelium (Fig. 4b, c, arrow). We found by H&E staining that LCO was shifted posteriorly, closer to the dorsal part of the left coronary sinus in the aortic root of E16.5 *Sox17*^*eKO*^ hearts (Fig. 4d, e). Furthermore, quantitative measurement for the distance from the coronary ostia to the base of the aortic valve (Fig. 4a, between lines *a’* and *b’*) revealed significant upward displacement of LCO (Fig. 4f), but not RCO (Supplementary Fig. 8a). Further correlation analysis between the underdeveloped NCL size and the upshift distance revealed that the severity of the two defects are significantly correlated (Supplementary Fig. 8b). To better visualize the upshifted LCO, we carried out a whole heart immunostaining for smooth muscle myosin heavy chain (smMHC), a definitive marker for mature VSMCs (Fig. 4g, h), and confirmed the high-takeoff LCO in the aortic root wall of E16.5 *Sox17*^*eKO*^ hearts (Fig. 4h, arrow). In addition, smMHC staining identified a tissue boundary between the smMHC-negative myocardium at the base of the aortic sinus and the smMHC-positive vascular smooth muscle covering the aortic sinus wall; noted that this tissue boundary was also upshifted in E16.5 *Sox17*^*eKO*^ hearts (Fig. 4h, double arrows). This muscular tissue boundary was already present in the young embryos collected at E13.5 as TnT immunostaining for the myocardium revealed a transition zone from the TnT-negative vascular smooth muscle to the TnT-positive myocardium in the control hearts, corresponding to the aortic sinus wall between the leading edge of the leaflets and the base of the aortic valve (Supplementary Fig. 9a). This transition was altered at this earlier stage (i.e., E13.5) as evidenced by the increased TnT-expressing cardiomyocytes in the LAS wall of *Sox17*^*eKO*^ hearts (Supplementary Fig. 9b). Furthermore, while the primitive LCO was seen in the LAS wall opening to the aortic lumen in the control heart (Supplementary Fig. 9a, arrowhead), only a rudiment vessel was found in the *Sox17*^*eKO*^ heart, which was located posteriorly within the LAS wall and remained unattached to the aortic lumen (Supplementary Fig. 9b, arrowhead). On the other hand, the primitive coronary stems were positioned normally in the *Sox17*^*eKO*^ heart as seen in the control (Supplementary Fig. 9a, b, arrow). These findings support that the SOX17-regulated formation of aortic root wall is critical for positioning LCO.

### Misplaced LCO reduces the coronary flow, leading to coronary anomalies

**Fig. 5:**
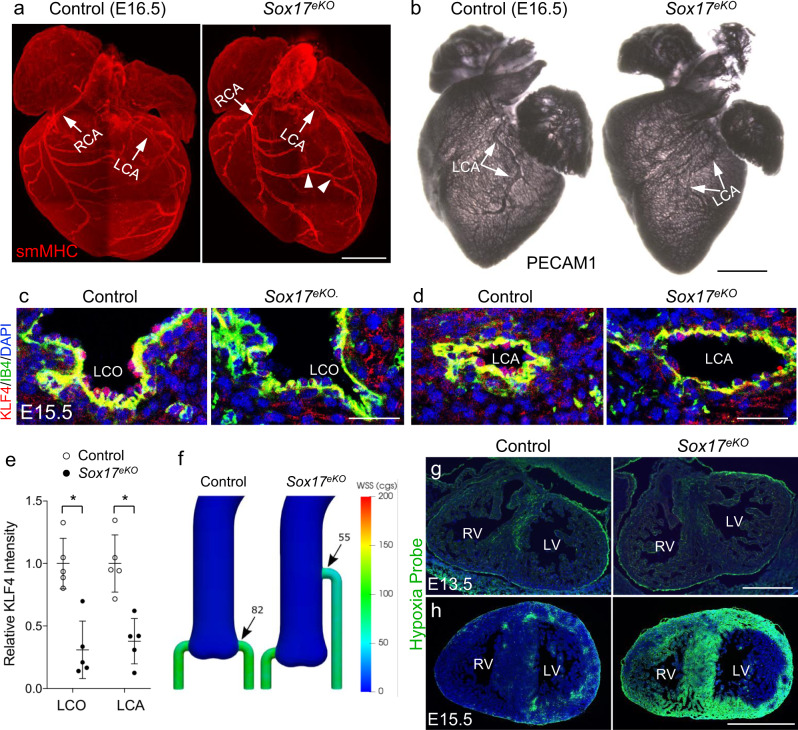
Misplaced LCO causes reduced coronary flow, leading to lethal anomalous coronary arteries. **a** Representative whole heart smMHC IF images (*n* = 5/group) of E16.5 control and *Sox17*^eKO^ hearts show narrowed LCA (arrow) and collateral vessels extended from RCA to LCA (arrowheads) in the mutant hearts. **b** Representative images of whole heart PECAM1 IF (*n* = 3/group) of E16.5 control and *Sox17*^eKO^ embryos show narrowed LCA (arrows) in *Sox17*^eKO^ hearts. **c**, **d** Representative IF images (*n*=5/group) of E16.5 control and *Sox17*^eKO^ hearts shows reduced endothelial expression of KLF4 (red, arrow) in LCO (**c**) and LCA (**d**) of *Sox17*^eKO^ hearts. **e** Quantitative analysis of (**c**, **d**) confirms reduced aortic-coronary flow at LCO. (*n* = 5/group, mean ± SD, unpaired two-tailed *t*-test, *p* = 0.001 for LCO, *p* = 0.0014 for LCA, *p < 0.05). **f** Computational simulation shows reduced wall sheer stress (WSS) from 82 to 55 cgs (centimetre–gram–second system of units) at LCO in E16.5 *Sox17*^*eKO*^ hearts. **g**, **h** Representative hypoxia probe staining images (*n* = 4/group) of E13.5 or E15.5 control and *Sox17*^eKO^ hearts shows severe hypoxia developed in the left ventricle (LV) of E15.5 *Sox17*^*eKO*^ hearts (**h**), which also affects the right ventricle (RV) after the establishment of coronary circulation around E14.5, but not E13.5 *Sox17*^*eKO*^ hearts before functional coronary circulation starts (**g**). Source data are provided as a Source Data file. Scale bars: 500 µm in **a**, **b**, **g**, **h**; 50 µm in **c**, **d**.

Misplaced coronary ostia is a major cause of CAAs as it can lead to insufficient coronary perfusion, myocardial ischemia and sudden cardiac death. We therefore investigated whether it might be the case for *Sox17*^*eKO*^ embryos. Whole mount immunofluorescence for smMHC revealed abnormal LCA in E16.5 *Sox17*^*eKO*^ hearts with a narrowed lumen (Fig. 5a, arrow) and overreaching branches or collateral vessels from the right coronary artery (RCA) interconnecting with LCA (Fig. 5a, arrowheads). Further whole mount immunostaining for PECAM1 confirmed the hypoplastic LCA in E16.5 *Sox17*^*eKO*^ hearts (Fig. 5b, arrows). The formation of collateral vessels would indicate a chronic myocardial hypoxia due to reduced coronary perfusion^52^. We therefore evaluated the impact of malformed LCO on the coronary perfusion using co-immunostaining for KLF4 (a molecular sensor of shear stress) and IB4 for endothelial cells. The staining results revealed that the endothelial KLF4 expression was markedly decreased at LCO (Fig. 5c, e) and LCA (Fig. 5d, e), supporting the reduced flow from the aorta to the LCA in the *Sox17*^*eKO*^ heart. This reduced blood flow was recapitulated by computational fluid dynamic (CFD) simulations using the scanning images from E16.5 control and *Sox17*^*eKO*^ hearts that showed a ~33% reduction in the wall shear stress (WSS) at misplaced LCO compared to normally positioned LCO (Fig. 5f). Consequently, the poor coronary perfusion resulted in myocardial hypoxia confirmed by the hypoxia probe staining for E15.5 *Sox17*^*eKO*^ hearts (Fig. 5g, h). Note that the right ventricle was also hypoxic due to the right to left shunting through the collateral vessels that stole the blood away from the right ventricle. The reduced coronary perfusion to both ventricles could explain the hypoplastic heart with poor compaction in both ventricles. Because myocardial hypoxia was not present in E13.5 *Sox17*^*eKO*^ hearts (Fig. 5g) before the establishment of the coronary circulation at E14.5, and the *Sox17* expression in the coronary vascular endothelium was not affected in the *Sox17*^*eKO*^ heart (Fig. 1b–d), anomalous coronary arteries and insufficient coronary perfusion were thus the direct result of misplaced LCO. Taken Together, these findings support that CAAs and BAV share the SOX17-dependent pathophysiology affecting the fate and functions of the SHF cell population in the aortic root. Furthermore, myocardial ischemia likely causes the hypoplastic heart, leading to the death of *Sox17*^*eKO*^ embryos.

### SOX17 regulates the expression of genes essential for aortic root development

To identify the SOX17-regulated genes involved in the aortic valve and coronary ostium defects, we performed transcriptomic (RNA-seq) analysis of the proximal OFT from E12.5 *Sox17*^*eKO*^ versus control hearts. We performed paired-end RNA-seq and applied DESeq2 to the gene read counts for differential expression analysis, using adjusted *p* value < 0.1 for statistical significance (see Supplementary Methods for details). The results showed that 412 protein-coding genes were upregulated and 148 were downregulated in the *Sox17*^*eKO*^ OFT (Supplementary Fig. 10a and Supplementary Data 1). We tested if these differential expressed genes (DEGs) contain the canonical SOX17 binding sites identified previously in mouse embryonic stem cells^53^ and found that these sites were enriched in both upregulated and downregulated DEGs, suggesting SOX17 binding in the OFT endothelial cells (Supplementary Fig. 10b and Supplementary Data 1). Further functional enrichment analyses of the DEGs showed that downregulated genes in the *Sox17*^*eKO*^ OFT were enriched for cell cycle, muscle/smooth muscle contraction and NOTCH signaling (Supplementary Fig. 10c and Supplementary Data 1), whereas upregulated genes were enriched in three biologically relevant categories: cell functions (communication, morphogenesis, adhesion and differentiation), cardiovascular development (aortic valve and aorta development) and PDGF signaling (Supplementary Fig. 10d and Supplementary Data 1). Of note, downregulated genes in smooth muscle contraction (*Cnn1*, *Cnn2*, *Myl9*) and upregulated genes in ECM (*Ctgf, Lama2/4*, *Fbn2*, *Vcan*) are consistent with the scenario of disrupted mesenchymal gene program impeding aortic root maturation. We validated the transcriptomic results by RT-qPCR using independent OFT samples, showing same changes in 13 of 16 DEGs (downregulated *Dcn*, *Col15a1, Sema3g, Gja4* and upregulated *Cnot3*, *Pkd2, Robo1*, *Tnfaip3, Hhex, Vcan, Angptl4, Pdgfra*, *Cdk6*) in the E12.5 *Sox17*^*eKO*^ OFT (Supplementary Fig. 10e, f). Downregulated NOTCH signaling in the *Sox17*^*eKO*^ OFT (Supplementary Fig. 10c) promoted us to examine the activated NOTCH1 in aortic root endothelium, which is critical for the development of aortic valve and coronary ostia^12,28,29^. We found that the level of NOTCH1 intracellular domain (N1ICD) was somewhat but not significantly reduced in the aortic root endothelium of *Sox17*^*eKO*^ hearts (Supplementary Fig. 10g). The modest decreased NOTCH signaling was unlikely to cause BAV due to the absence of NCL as the N1ICD inactivation in aortic root endocardium using *Nfatc1*^*enCre*^ results in a different type of BAV due to the fusion of RCL and LCL.

### SOX17 regulates aortic valve development through the PDGF signaling

**Fig. 6:**
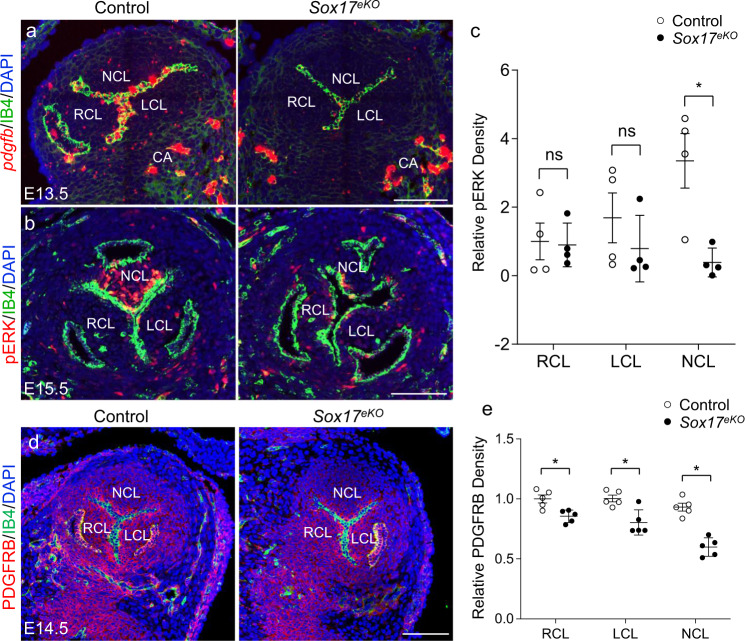
SOX17 regulates PDGFB signaling in the NCL mesenchyme. **a** Representative RNAscope ISH images (*n* = 3/group) of E13.5 control and *Sox17*^*eKO*^ hearts show diminished *Pdgfb* expression (red) in the aortic valve endothelium in *Sox17*^*eKO*^ hearts. Noted that only the large dots are considered the real signals. **b**, **c** IF images of E15.5 control and *Sox17*^*eKO*^ hearts show significantly reduced phosphorylated ERK (pERK) density (red) in the NCL mesenchyme of *Sox17*^*eKO*^ hearts. (*n* = 4/group, mean ± SD, unpaired two-tailed *t*-test, *p* = 0.011, *p < 0.05). **d**, **e** IF images of E14.5 control and *Sox17*^*eKO*^ hearts show significantly reduced PDGFRB density (red) in the aortic valve region, especially in NCL, of the *Sox17*^*eKO*^ hearts. (*n* = 5/group, mean ± SD, unpaired two-tailed *t*-test, *p* = 0.007 for RCL, *p* = 0.006 for LCL, *p* = 0.0001 for NCL, *p < 0.05). Source data are provided as a Source Data file. Scale bars: 100 µm.

We therefore focused on the PDGF signaling, because PDGFB is a major vascular growth factor produced by the vascular endothelium to support the proliferation of adjacent mesenchyme^54^ and previous studies have shown that endothelial PDGFB is required for cardiac and vascular development^55–57^. RNAscope ISH revealed that the expression of *Pdgfb* was specifically abolished in aortic valve endothelium of E13.5 *Sox17*^*eKO*^ heart, whereas its expression in coronary arterial endothelium was not affected (Fig. 6a). Consistently, immunostaining showed a high level of pERK (downstream target of PDGF signaling) predominantly present in the NCL mesenchyme of the control E15.5 heart, and such expression was markedly diminished in the *Sox17*^*eKO*^ heart (Fig. 6b, c) while the total ERK expression in the NCL was only minimally but not significantly affected (Supplementary Fig. 11a). Furthermore, immunostaining of PDGFRB, a main receptor for PDGFB, showed a preferred expression in the aortic valve mesenchyme, and such expression was significantly reduced in the *Sox17*^*eKO*^ heart (Fig. 6d, e). In addition, we found the expression of PDGFRA in the aortic sinus endothelium (Supplementary Fig. 11b). Altogether, these observations support a role for SOX17-dependent PDGFB growth signaling to promote the NCL growth, likely through PDGFRB.

**Fig. 7:**
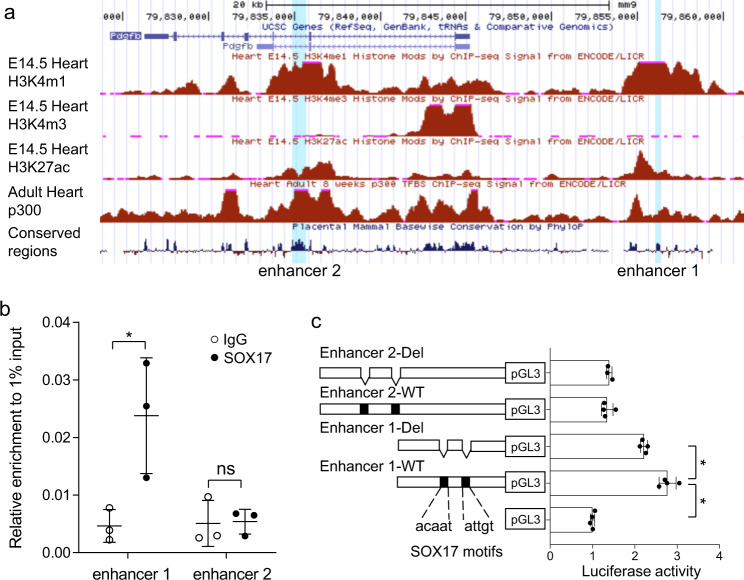
SOX17 regulates Pdgfb expression by directly binding to Pdgfb distal enhancer. **a** UCSC Genome browser tracks show two *Pdgfb* transcriptional enhancers containing the consensus SOX17 binding motifs, one located at the ~10 Kb upstream of the first exon of *Pdgfb* (enhancer 1) and the other between exon 2 and 3 (enhancer 2) identified for E14.5 and adult mouse hearts based on the mouse ENCODE project. **b** ChIP-qPCR shows the binding of SOX17 at enhancer 1. (*n* = 3/group, mean ± SD, unpaired two-tailed *t*-test, *p* = 0.03 for enhancer 1, *p < 0.05). **c** Reporter gene assays show that enhancer 1 activates the promoter of reporter gene and the mutagenesis analysis indicates that the activation is dependent on the intact SOX17 binding motifs. (*n* = 4/group, mean ± SD, unpaired two-tailed *t*-test, *p* = 0.000003 for enhancer 1-WT, *p* = 0.002 for enhancer 1-Del, *p < 0.05). Source data are provided as a Source Data file.

Two transcriptional enhancers containing the consensus SOX17 binding motifs were identified for *Pdgfb* in the E14.5 and 8-week old mouse heart using data from the mouse ENCODE project^58^; one is located at ~10 kb upstream regulatory region of the gene (enhancer 1) and the other is in the gene body (enhancer 2) (Fig. 7a). To determine whether SOX17 binds to the two *Pdgfb* enhancers, we performed ChIP-qPCR using the mouse cardiac endothelial cells (MCECs), which were used to obtain sufficient number of cells for this assay. The result showed the binding of SOX17 to the enhancer 1, but not the enhancer 2 (Fig. 7b). Consistent with the ChIP-qPCR data, reporter gene assays for the wild type (WT) and the mutated enhancers without the SOX17 binding motifs showed that the enhancer 1, but not the enhancer 2, was able to activate the promoter of the reporter gene in the cultured MCECs, and the activation was dependent on the intact SOX17 binding motifs (Fig. 7c). In addition to the SOX17 binding sites, conservation analysis using sequence alignment by ClustalW^59^ revealed that the *Pdgfb* enhancer 1 is situated in a region highly conserved among mouse, rat, rhesus, and human sequences and containing the binding motifs for SP1/SP3^60^, SMAD^61^, and ELK1^62^ which have been suggested for regulating the *Pdgfb* transcription (Supplementary Fig. 12). Together, these observations suggest that *Pdgfb* is a direct target of SOX17 in aortic root endothelium that mediates the SOX17-dependent aortic root development.

### PDGFB acts downstream of SOX17 to regulate aortic root development

**Fig. 8:**
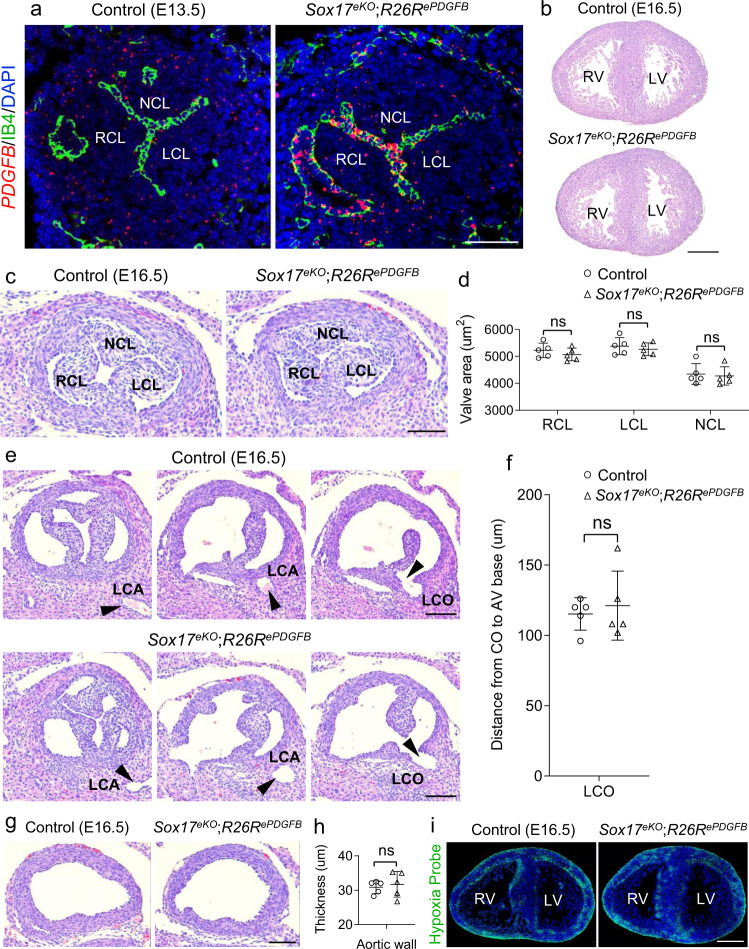
PDGFB re-expression rescues aortic root defects. **a** Representative RNAscope images (*n* = 3/group) of E13.5 control and *Sox17*^*eKO*^*;R26R*^*ePDGFB*^ hearts showing the human *PDGFB* expression (red) in aortic endothelium in the *Sox17*^*eKO*^*;R26R*^*ePDGFB*^ heart. **b**, **c** H&E staining (*n*=5/group) showing normal ventricular wall and NCL, respectively, in all 5 E16.5 *Sox17*^*eKO*^*;R26R*^*ePDGFB*^ embryos examined. LV/RV: left and right ventricle. **d** Quantitative analysis shows comparable NCL area in E16.5 *Sox17*^*eKO*^*;R26R*^*ePDGFB*^ and control hearts. *n* = 5/group, mean ± SD, unpaired two-tailed *t*-test, ns, no significance. **e** Representative H&E stained continuous sections E16.5 control and *Sox17*^*eKO*^*;R26R*^*ePDGFB*^ hearts from the base of aortic valve leaflets to the coronary ostia showing that LCO is correctly positioned in E16.5 *Sox17*^*eKO*^*;R26R*^*ePDGFB*^ hearts compared to the controls. Arrowheads indicate the main coronary arteries and ostia. **f** Quantitative analysis showing no difference in the LCO position related to aortic valve base between *Sox17*^*eKO*^*;R26R*^*ePDGFB*^ hearts and controls. *n* = 5/group, mean ± SD, unpaired two-tailed *t*-test, ns, no significance. **g**, **h** H&E staining and quantitative analysis show the normal aortic wall thickness in E16.5 *Sox17*^*eKO*^*;R26R*^*ePDGFB*^ hearts. *n* = 5/group, mean ± SD, unpaired two-tailed *t*-test, ns, no significance. **i** Representative hypoxia probe staining images (*n* = 5/group) showing the similar staining pattern and intensity between E16.5 control and *Sox17*^*eKO*^;R26P hearts. Source data are provided as a Source Data file. Scale bars: 100 µm in **a**, **c**, **e**, **g**; 200 µm in **b**, **i**.

**Fig. 9:**
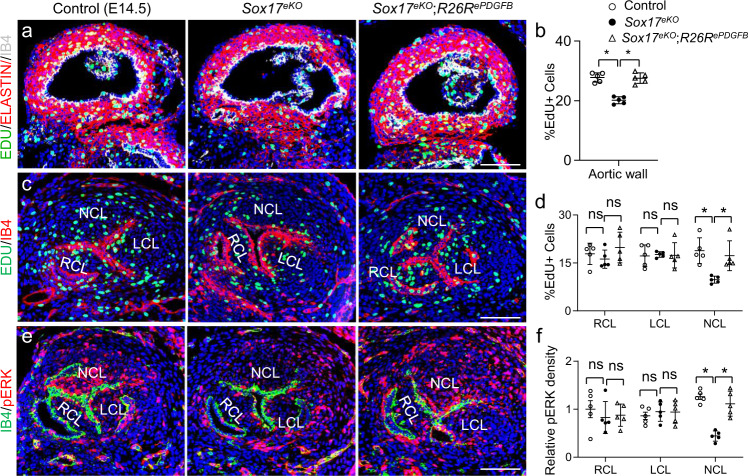
PDGFB re-expression rescues reduced cell proliferation of VSMCs and NCL and decreased pERK activity resulted from the Sox17 deletion. **a**, **b** EdU (green) assay and quantitative analysis showing the reduced proliferation of VSMCs in the aortic root wall marked by ELSTAIN (red) staining in the *Sox17*^*eKO*^ hearts was rescued in the *Sox17*^*eKO*^*;R26R*^*ePDGFB*^ heart. IB4 (white) stains the endothelium. *n* = 5/group, mean ± SD, one-way ANOVA Tukey’s test, *p* = 0.00002 for *Sox17*^*eKO*^, *p* = 0.00006 for *Sox17*^*eKO*^*;R26R*^*ePDGFB*^, *p < 0.05). **c**, **d** EdU (green) assay and quantitative analysis showing the reduced proliferation of NCL in the *Sox17*^*eKO*^ hearts was rescued in the *Sox17*^*eKO*^*;R26R*^*ePDGFB*^ hearts. *n* = 5/group, mean ± SD, one-way ANOVA Tukey’s test, *p* = 0.001 for NCL of *Sox17*^*eKO*^, *p* = 0.008 for NCL of *Sox17*^*eKO*^*;R26R*^*ePDGFB*^, *p < 0.05. **e**, **f** IF images and quantitative analysis showing the reduced phosphorylated ERK (pERK) density (red) in the NCL mesenchyme of the *Sox17*^*eKO*^ hearts was restored in the *Sox17*^*eKO*^*;R26R*^*ePDGFB*^ heart. *n* = 5/group, mean ± SD, one-way ANOVA Tukey’s test, *p* = 0.000004 for NCL of *Sox17*^*eKO*^, *p* = 0.0006 for NCL of *Sox17*^*eKO*^*;R26R*^*ePDGFB*^, *p < 0.05. Source data are provided as a Source Data file. Scale bars: 100 µm.

To directly test the SOX17-PDGFB functional axis in aortic root endothelium for aortic root development, we carried out the genetic rescue experiment by restoring the PDGFB expression in aortic root endothelium of the *Sox17*^*eKO*^ heart using the *Nfatc1*^*enCre*^ and *R26R*^*fshPDGFB*^ mice^63^. We first examined E17.5 *Nfatc1*^*enCre*^*;R26R*^*fshPDGFB*^ embryos (*R26R*^*ePDGFB*^ hereafter) and found that some of them are relatively underdeveloped (Supplementary Fig. 13a). We then analyzed the heart of E16.5 *R26R*^*ePDGFB*^ embryo and did not notice obvious defects in the ventricle (Supplementary Fig. 13b), aortic valve (Supplementary Fig. 13c, d), and LCO (Supplementary Fig. 13e, f). Furthermore, we did not observe myocardial hypoxia in E17.5 *R26R*^*ePDGFB*^ heart (Supplementary Fig. 13g) which has normal coronary vessels (Supplementary Fig. 13h). Not all *R26R*^*ePDGFB*^ embryos were viable at birth, but we were able to carry out the rescue experiments as more than half of them survived to E16.5 when the rescued embryos were examined. After crossing the *R26R*^*ePDGFB*^ mice to the *Sox17*^*eKO*^ background, we confirmed using RNAscope ISH probe specific for the human *PDGFB* (but not the mouse) transcripts that they were specifically expressed in aortic valve endothelium of E13.5 *Sox17*^*eKO*^*;R26R*^*fshPDGFB*^ hearts (*Sox17*^*eKO*^*;R26R*^*ePDGFB*^ hereafter) as no RNAscope signals were found in the control hearts (*Sox17*^*f/+*^*;R26R*^*fshPDGFB*^ or *Sox17*^*f/f*^*;R26R*^*fshPDGFB*^) (Fig. 8a). We found by H&E staining of E16.5 hearts from the control and *Sox17*^*eKO*^*;R26R*^*ePDGFB*^ embryos that the restoration of PDGFB expression was able to rescue the hypoplastic heart resulted from the *Sox17* deletion (comparing Fig. 8b to Fig. 1e) and importantly, none of 16 *Sox17*^*eKO*^*;R26R*^*ePDGFB*^ hearts examined had the NCL defect or developed BAV (Fig. 8c, d). The restoration of PDGFB expression also rescued the misplaced LCO in E16.5 *Sox17*^*eKO*^ hearts (Fig. 4d–f) as it positioned correctly in the aortic root of the *Sox17*^*eKO*^*;R26R*^*ePDGFB*^ heart (Fig. 8e, f). Furthermore, the thin aortic root wall (Fig. 3a, b) and the defective coronary arteries of the *Sox17*^*eKO*^ heart (Fig. 5a, b) were all recovered in the *Sox17*^*eKO*^*;R26R*^*ePDGFB*^ heart (Fig. 8g, h and Supplementary Fig. 14, respectively), and comparable myocardial hypoxia was found between the *Sox17*^*eKO*^*;R26R*^*ePDGFB*^ and the control heart (Fig. 8i). On the cellular level, the restoration of PDGFB expression reversed the proliferation of VSMCs and NCL mesenchymal cells in the *Sox17*^*eKO*^*;R26R*^*ePDGFB*^ heart to the normal level (Fig. 9a–d). Mechanistically, the expression of pERK was restored in the NCL of the *Sox17*^*eKO*^*;R26R*^*ePDGFB*^ heart to the level was comparable to the control heart (Fig. 9e, f). Although the aortic root phenotypes and PDGF signaling were improved substantially in the *Sox17*^*eKO*^*;R26R*^*ePDGFB*^ embryos and they were able to survival to E18.5 while all the *Sox17*^*eKO*^ embryos were dead before E18.5, few of *Sox17*^*eKO*^*;R26R*^*ePDGFB*^ neonates were found at birth. These observations support, at least, partial rescue of the aortic valve growth defect by the restoration of the PDGFB-pERK signaling in NCL via expressing human PDGFB in the aortic root endothelium. However, the pERK signaling is prolonged in the rescued hearts (Supplementary Fig. 15), which might be detrimental to late heart valve maturation and/or functions impacting on survive. Altogether, the results from the morphological, cellular and molecular signaling studies support that the reduced expression of PDGFB in aortic root endothelium of *Sox17*^*eKO*^ embryos is a major disease mechanism causing decreased proliferation of aortic root cells derived from SHF, resulting in complex aortic root defects, i.e., BAV without NCL and misplaced LCO, that lead to reduced coronary perfusion, myocardial hypoxia, hypoplastic ventricles and ultimately, the death of the embryos.

## Discussion

**Fig. 10:**
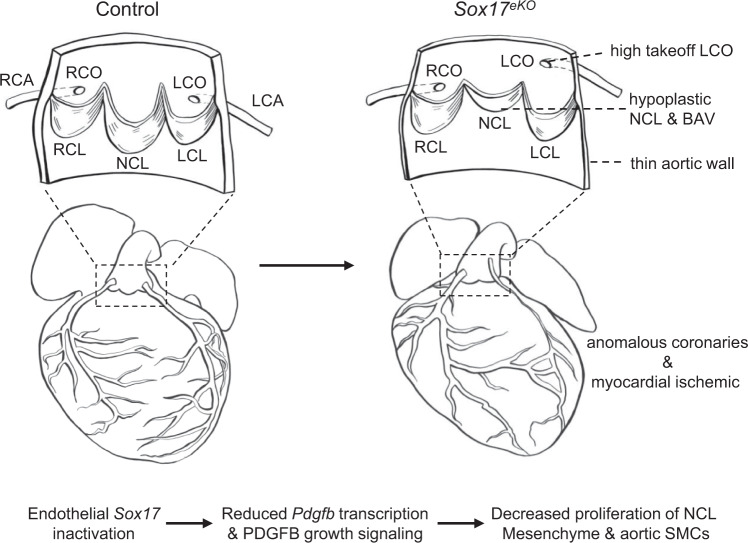
Summary of molecular, cellular and tissue structural changes in the mouse model of BAV syndrome caused by the Sox17 inactivation in aortic root endothelium. A working model showing the SOX17 deficiency results in downregulated *Pdgfb* transcription in aortic root endothelium and reduced endothelial to mesenchymal PDGFB signaling that lead to cell proliferation and maturation defects in the developing aortic root. These defects cause complex aortic root malformations including BAV, hypoplastic aortic wall, as well as high takeoff left coronary ostium that lead to lethal coronary artery anomalies.

In this study, by deleting *Sox17* in aortic root endothelium in mice and analyzing the resultant cardiac phenotypes, we show that *Sox17* is required for aortic valve development and coronary ostium patterning (Fig. 10). We also elucidate an essential role of SOX17 in regulating proliferation and differentiation of the NCL mesenchyme and aortic root smooth muscle derived from the SHF cardiomyocytes. By analyzing the altered developmental course, we present a morphological link between the anomalous NCL and misplaced LCO. By further analyzing gene expression and regulation, we uncover a disease mechanism involving PDGF signaling by which the SOX17 deficiency alters the essential growth signal during aortic root development. Finally, we establish by the genetic rescue approach a SOX17-PDGFB signaling axis critical for aortic root development, informing that the disruption of this regulatory axis, such as mutations in the genes involved in the signaling axis, may cause clinically significant congenital aortic root defects.

SOX17 belongs to the conserved SOX F family (including *Sox7*, *Sox17* and *Sox18*) of transcription factors and participates in the differentiation of definitive endoderm, hematopoietic cells, and vasculogenesis^34,64^. Previous studies have shown SOX F transcription factors cooperatively regulate cardiovascular development^65–68^. In this study, we found that the expression of SOX7 and *Sox18* in the aortic root region was not altered in the *Sox17*^*eKO*^ heart by immunostaining and RT-qPCR, respectively (Supplementary Fig. 16). Importantly, our results indicate that the loss of *Sox17* cannot be compensated by *Sox7* and/or *Sox18*. However, we cannot exclude the possibility that the complementary effect may occur in other heart valves (such as tricuspid valve, mitral valve and pulmonary valve) to protect their normal development from the loss of *Sox17*. In the embryonic vasculature, *Sox17* is selectively expressed in the arterial endothelial cells^30,69,70^. Endothelial-specific inactivation of *Sox17* in mice leads to impaired arterial specification and embryonic death with heart defects^30^, whereas conditional deletion of *Sox17* in the cardiac progenitor cells causes cardiac chamber defects with reduced endocardial cell proliferation^71^. Of note, a recent study has reported the essential role of *Sox17* in controlling coronary artery emergence and development^72^. During coronary artery development, the NOTCH signaling also plays an indispensable role^73–75^ and several studies have demonstrated interactions between SOX17 and the NOTCH signaling^30,31,76^. While these interactions are within the artery progenitor or artery endothelial cells, the aortic root endothelium specific function of SOX17 remains unknown, where the deletion of *Notch1* or *Sox17* results in distinct BAV subtypes in the *Notch1*^*eKO*^ (fusion of LCL and RCL)^28^ or *Sox17*^*eKO*^ (loss of NCL) mouse embryo. These observations suggest different mechanisms underlying the developmental defects regulated by NOTCH1 and SOX17 independently. In support of this notion, the cellular phenotypes in the two BAVs are different as BAV observed in the *Notch1*^eKO^ embryo is associated with reduced apoptosis of LCL and RCL mesenchymal cells^28^ and increased proliferation of valve endothelial cells^28,29^, whereas BAV in the *Sox17*^eKO^ embryo is associated with reduced proliferation of NCL mesenchyme. The notion is further supported by the fact that we did not observe significant changes in the levels of NICD in the aortic valve endothelium after the *Sox17* deletion (Supplementary Fig 10g).

Unlike LCL and RCL, which receive the mesenchymal cells by EndMT^35,38,39^ and from NCCs^41–43^, NCL is made of mesenchymal cells largely from the SHF^44,45,49^. The different embryonic origins of aortic valve leaflets are likely responsible for the distinct BAV subtypes when cell fate and/or function of individual lineages are disrupted by a cell-type specific mechanism. For instance, inactivation of *Notch1* in aortic valve endothelium^28,29^ or *Gata6* in SHF^21^ in mice causes a BAV due to the fusion of LCL and RCL, which resembles the most common form of BAV in humans^77,78^, whereas mice with loss of *Gata5* in the endocardium/endothelium, *Fgf8* or *Krox20* in NCCs display BAV resulted from the fusion of NCL and RCL^18,42,79^, a relatively less common form of BAV. In contrast, BAV resulting from the absence of NCL is rare and associated with severe cardiac complications^77,78^. Consistently, our mouse study shows that the absence of NCL is embryonic lethal, owing to severe coronary artery complications. A similar NCL phenotype has been reported in the BAV model when the NOTCH1-JAG1 signaling is disrupted in the SHF progenitors^44^ or endothelial NOS3 is inactivated^80^. In our *Sox17*^*eKO*^ model, the SHF progenitors specified for the NCL are unlikely affected as our *Nfatc1*^*enCre*^ mediated *Sox17* deletion begins after E11.5 and the SHF progenitors are already colonized NCL before E11.5. Rather, our data would support the failure in the proliferation of the SHF-derived NCL mesenchyme. We suggest that the SHF origin of the NCL might be a reason that NCL was specifically affected, as SHF did not have a significant contribution to RCL and LCL. However, this idea needs to be further explored experimentally. Nonetheless, these observations support a complex signaling network among multiple cell lineages that govern the NCL formation.

We propose that the SOX17-PDGFB axis is part of this signaling network, as the expression of *Pdgfb* is dependent on SOX17 in aortic root endothelium and the proper NCL formation requires the axis, although we cannot exclude other signals such as the FGF signaling in the network. In support of the SOX17-PDGF functional axis, knockout mice for either *Pdgfb* or *Pdgfra* have multiple cardiac and coronary vascular defects^55,56,81^, and *Pdgfb* global nulls and endothelial knockout mice display underdeveloped valves and abnormal coronary ostia^55,57^. These abnormalities are similar to the defects found in the *Sox17* nulls where the high level of PDGFB signaling, as shown by pERK expression, is nearly abolished in NCL. PDGFB secreted from aortic valve endothelium is expected to promote the NCL mesenchymal proliferation by activating ERK. Indeed, reactivating ERK by the expression of human PDGFB in aortic valve endothelium is able to rescue the NCL phenotype in the *Sox17* nulls. Whether the same mechanism promotes the proliferation of aortic root smooth muscle cells is less clear from our study. Nonetheless, the well-known critical role of PDGF signaling in the recruitment and proliferation of VSMCs during development support that the SOX17 dependent PDGFB signaling also operates in aortic endothelium for aortic root smooth muscle growth. Additionally, Inactivation of *Sox17* affects the expression of ECM genes, such as *Ctgf*, involving aortic root maturation that are not fully investigated in this study, future studies will focus on how SOX17 regulates the ECM synthesis during aortic root maturation.

Another important observation is that proper aortic root development is essential for normal patterning of coronary ostia^82^. In this regard, BAV is associated with coronary ostium anomalies in humans^5–7^. Similarly, there is a high incidence of anomalous coronary ostia in animal models with BAV^83^. Previous mouse studies have shown that coronary ostia are formed as the endothelial cells from the peritruncal capillaries (the primitive coronary vessels around the aortic root) penetrate the aortic wall by localized apoptosis^84–86^. When the coronary circulation starts, the VSMCs are recruited to the coronary ostia and then, progressively, more distally to the main coronary stems, to stabilize the vessels in a high shear stress environment^87,88^. In support of this in-growth of the coronary vasculature as a major developmental mechanism to form the coronary ostia, we show that the aortic and the coronary endothelial cells form a clear tissue boundary at the coronary ostia and the aortic endothelial cells do not outgrow into the main coronary arteries. VEGF-C and ISL1 have been suggested to guide the connection between the coronary arteries and the aorta^89^. We, therefore, examined the VEGFC expression in our *Sox17*^*eKO*^ model and found that the VEGFC expression pattern and level in the cardiomyocytes are not affected by the *Sox17* deletion (Supplementary Fig. 17). Similarly, the ISL1 expression in the cardiomyocytes is not altered in the *Sox17*^*eKO*^ heart (Supplementary Fig. 7c, e). Considering the disruption of *Sox17* in the aortic endothelium leads to the high-takeoff LCO but not RCO, we propose that SOX17 works as an intrinsic factor, like TBX1 which has been demonstrated required for the patterning of coronary artery stems and the formation of a leftward positioned coronary ostium, to regulate specifically LCO formation^86^. Clinical studies have also suggested that the high takeoff LCO occurs more frequently in children with BAV and associated congenital heart disease^7^. Taken together, these observations support a complex signaling network needed to be further discovered that governs the CO formation.

Anomalous coronary ostia can lead to CAAs, so do intrinsic coronary artery defects. It is difficult to assess whether CAAs are the direct consequence of genetic defects or secondary to disrupted flow dynamics. In our study, using a tissue-specific genetic deletion that spares the coronary arteries, we are able to show that anomalous coronary ostia cause CAAs, leading to myocardial hypoxia and embryonic death. Our mouse model allows the investigation of flow-dependent molecular mechanisms underlying coronary artery maturation and its interactions with ventricular compaction and maturation in utero.

## Methods

### Animal models

Mice were used in this study. Mouse housing and experiments were performed according to the protocol approved by the Institutional Animal Care and Use Committee of Albert Einstein College of Medicine. Mice were maintained under specific pathogen-free conditions in mouse rooms under a 12 h–12 h light–dark cycle at 22 °C and under ~55% humidity. Mice received sterile autoclaved water and a standard diet. The aortic root endothelial *Sox17* knockout mice were generated by crossing the floxed *Sox17* mice^34^ with the transgenic *Nfatc1* enhancer Cre mice^35^. The aortic root endothelial *human PDGFB* simultaneous overexpression in *Sox17*^*eKO*^ mice were generated by crossing the floxed *Sox17* and *R26R*^*fshPDGFB*^ mice^63^, obtained from Dr. Christer Betsholtz, with the *Nfatc1*^*enCre*^*:Sox17*^*f/+*^ mice. The transgenic TnT-Cre (*TnT*^*Cre*^) mouse^50^ and the *R26R*^*fsGFP*^ reporter mouse^51^ were used in this study. All mice were maintained on the C57BL/6J background. All mouse experiments were carried out on the embryos isolated from the pregnant female mice which were sacrificed immediately after anesthesia after inhaling isoflurane. Embryos were isolated and inspected according to expected developmental ages, underdeveloped embryos were excluded from studies. Embryos from the same little were used for subsequent experiments. Noontime on the day of detecting vaginal plugs was designated as E0.5. The yolk sac or tail of embryo was used for PCR genotyping.

### Histology and morphology analyses

Hematoxylin and eosin (H&E) staining was performed on serial sections (6 μm) of the entire hearts to evaluate the histology and morphology of the heart chambers and heart valves. 3D reconstruction of aortic valves from E16.5 *Sox17*^*eKO*^ embryos and control littermates were performed by digital photographs of H&E stained serial sections.

### Immunofluorescence

The preparation of paraffin and frozen sections and immunostaining were carried out following standard protocol as described previously^74^. For paraffin sections, embryonic hearts were freshly isolated in PBS, fixed in 4% PFA at 4 °C for 2 h to overnight according to developmental stages, washed in PBS, dehydrated through a serial of gradient ethanol, cleared in xylene and embedded in paraffin. The hearts were then sectioned at 6 μm thickness and the tissue sections were mounted on positive charged slides. H&E staining was performed using the standard protocol to evaluate the histology and pathology of the heart. For frozen sections, the hearts were isolated freshly, fixed in 4% PFA at 4 °C for 2 h to overnight depending on the size of the tissues, soak in 15% and 30% sucrose sequentially and embedded in the OCT compound with orientation for front or transverse sections. The hearts were then sectioned at 8 μm thickness, the tissue sections were mounted on positive charged slides, post-fixed in cold ethanol and acetone (1:1) solution for 5 min and stored at −80 °C. Tissue sections were air dried for 45 min before immunostaining by blocking with 5% normal horse or goat serum for 1 h at room temperature (RT) and incubating with primary antibodies in blocking buffer at 4 °C overnight, and then incubated with secondary antibodies conjugated with the Alexa 568 or 488 fluorescence dyes for 1 h at RT. Antibody information was listed in Supplementary material, Supplementary Table 2. Images were taken by a Zeiss Observer Z1 or Leica SP5 confocal microscope. All imaging quantifications were carried out blindly using Image J.

### Whole mount immunofluorescence staining

To visualize coronary ostium and coronary vessels in the embryonic hearts, whole mount staining was performed using antibodies against smMHC and PECAM1. The hearts were isolated from E16.5 control and Sox17^eKO^ embryos and fixed in 4% PFA for 3 h at 4 °C. The fixed hearts were blocked in 5% normal horse serum for 3 h at 4 °C and followed by incubation with primary antibodies at 4 °C overnight. The hearts were then washed for five times with PBS containing 0.5% TritonX-100 and incubated with biotinylated secondary antibodies or secondary antibodies conjugated with the Alexa 568 or 488 fluorescence dyes at 4 °C overnight. The hearts were then washed for five times with PBS containing 0.5% TritonX-100. For biotinylated secondary antibodies, the hearts were incubated with streptavidin from ABC Kit (Vector Laboratories) at 4 °C overnight. The hearts were then washed for five times with PBS containing 0.5% TritonX-100 and developed color using DAB Kit (Vector Laboratories). The stained hearts were photographed using a Leica confocal microscope. For secondary antibodies conjugated with the Alexa 568 or 488 fluorescence dyes, the hearts were cleared using (1-part benzyl alcohol, 2-part benzyl benzoate)^90^ before imaged using a Leica SP5 confocal microscope.

### Cell proliferation and apoptosis assays

Cell proliferation and cell apoptosis were determined using EdU and TUNEL assays, respectively, as described previously^28^. For cell proliferation assay, pregnant female mice were injected with EdU (Life Technology) through IP at a concentration of 100 mg/kg. After a 2 h pulse, the hearts were collected and processed for frozen sections as described above in the Immunostaining Section. The serial sections crossing the whole heart were first stained with IB4 or PECAM1 and ELASTIN antibody followed by EdU staining with EdU imaging Kit (Life Technology) and counterstained with DAPI (Vector lab). The stained sections were photographed using a Zeiss Observer Z1 or Leica SP5 confocal microscope. EdU-positive cells were counted using Image J and the data was presented as the ratio of EdU-positive cells among total cells. Three hearts were analyzed for each genotype. Apoptotic cells in E14.5 hearts of embryos were visualized by TUNEL assay. The frozen sections of isolated hearts were prepared as described in the EdU assay. Serial sections were first stained with IB4 or PECAM1 and ELASTIN antibody, followed by TUNEL assay using DeadEnd^™^ Fluorometric TUNEL System (Promega) and counterstained with DAPI. The stained sections were photographed using a Zeiss Observer Z1 or Leica SP5 confocal microscope.

### Three-dimensional (3D) reconstruction of aortic valve

Amira^TM^ software (version 6.1.1, FEI, Hillsboro, OR) was used for 3D reconstruction of aortic valves from E16.5 Sox17^eKO^ embryos and control littermates. Serial sections (6 μm) were sectioned from paraffin-embedded samples and stained with H&E. Digital photographs of heart sections were entered sequentially into Amira and aligned using the sum of least squares alignment algorithm. Additional alignment corrections were made visually by using the outline of the aortic wall as landmark.

### Detection of hypoxia

Myocardial hypoxia was detected using the Hypoxyprobe Kit (Hypoxyprobe Inc). The pregnant female mice were injected with the Hypoxyprobe through intraperitoneal (IP) at a concentration of 60 mg/kg body weight. After 90 min pulse, the hearts were isolated and processed for frozen section as described above. Then the hypoxic tissue was detected by immunostaining of the Hypoxyprobe antibodies and the Alexa Fluor 488 goat anti-mouse IgG secondary antibodies (Thermo Fisher Scientific, A28175, 1:200). The stained tissues were photographed using a Zeiss Observer Z1 microscope or Leica SP5 confocal microscope.

### Measurement of aortic blood flow by echocardiography

Echocardiography was performed using a Vevo 770 ultrasound with a real time micro-visualization transducer to evaluate the aortic blood flow velocity in E16.5 control and *Sox17*^*eKO*^ littermates. Pregnant female mice were anesthetized by inhalation of 2% isoflurane in glass chamber and 1–1.5% isoflurane via a nose cone. Investigators were blinded to genotypes of mice and subsequent data analysis.

### Coronary flow simulation analyses

Coronary flow was analyzed by computing simulation based on the whole mount images from E16.5 hearts. Briefly, the shear rate is on the order of 1/s throughout much of the simulation. However, it can reach values more than an order of magnitude larger, particularly in the LCO. Thus, a non-Newtonian viscosity model was used to ensure that differences in viscosity stemming from this range of shear rates are captured. Specifically, the Carreau model was used to model the dependence of the viscosity on the shear rate. Data of viscosity of adult mice^91^ was used to fit the constants in this equation in a least-squares sense, resulting in a zero-shear viscosity of 1.38 × 10^−2 ^Pa.s and an infinite-shear viscosity of 3.03 × 10^−3 ^Pa.s. A density of 1060 kg/m^3^ was used. The numerical time step size is set to 10^−4^s to ensure stability and accuracy of the simulations. Note that, as the viscosity data of adult mice is used, the non-Newtonian effect is expected to be stronger and the viscosity higher than would occur in embryonic mice due to the increased hematocrit. In vivo geometry (diameters, lengths) of the aortic root, coronary positions, and vascular lumens were directly traced at each section of the Z-stack (~ 2 μm voxel resolution, >100 images per embryo). Given the known variability of coronary patterning in the mouse heart, we focused on the primary hemodynamic consequences of differential aortic root and coronary ostia/entry lengths in diastole by truncating the coronary geometry at the border of the ventricular myocardium. We applied a modified computational models of discretized volumetrically with 1.5 × 10^5^ tetrahedral elements using TetGen^92^. The 3D incompressible flow equations are solved using an in-house, stabilized finite element solver. The governing equations are discretized with P1 finite elements in space and using the Generalized-*α* method in time. The system of linear equations is solved using the bi-partitioned method that includes a specialized preconditioner and an efficient parallel data structure algorithm. This code has been validated and used in multiple computational hemodynamics scenarios^93–96^. Non-linear iterations continued until the normalized residual was <10^−3^. Reducing the residual below this level did not have any significant effect on the results. A minimally intrusive stabilization method was employed to avoid rapid simulation divergence in case of backflow at Neumann boundaries^97^, and a multi-domain approach to handle resistance boundary conditions (BC)^98^. Due to the shear-thinning nature of blood, the fluid was treated as a non-Newtonian fluid. This non-Newtonian behavior was modeled using the Carreau model. This model has an asymptotic viscosity at low and high shear rates, with power-law behavior between these asymptotes. This model is described by the equation

$${\mu }_{{eff}}={\mu }_{{{\infty }}}+\left({\mu }_{0}-{\mu }_{{{\infty }}}\right){\left(1+{\left(\lambda \dot{\gamma }\right)}^{\alpha }\right)}^{(n-1)/\alpha }$$

and was used for its good fit with empirical data over the ranges of shear rates present in the simulations, where ${\mu }_{\infty }$ is the infinite-shear viscosity, ${\mu }_{0}$ is the zero-shear viscosity, $\lambda$ is the relaxation time, $\alpha$ is a smoothing parameter, $n$ is the power-law exponent, and $\dot{\gamma }$ is the shear rate. The shear rate is calculated as $\dot{\gamma }=\sqrt{{{{{{\boldsymbol{E}}}}}}:{{{{{\boldsymbol{E}}}}}}}$, where ${{{{{\boldsymbol{E}}}}}}$ is the strain rate tensor. Since the Womersley number is of *0* (10^−3^), suggesting the transient nature of the flow is insignificant, we made use of quasi-steady assumption to compute BCs. Since the coronaries are primarily perfused during diastole, the BCs are selected to model only this part of the cardiac cycle. For the aortic wall, we imposed a Dirichlet condition with an inward wall-normal velocity of 0.33 mm/s that is computed assuming 10% contraction during diastole that is 30% of the cardiac cycle^99^, a heart rate of 240 beats per minutes, and an equivalent aortic diameter of 0.5 mm^100^. A constant pressure BC of 9 mmHg is imposed at the aortic outlet^101^. A resistance boundary condition is imposed at the coronary outlets based on the coronary flow rate a s a percentage of the total cardiac output and diastolic pressure, an equivalent vascular resistance of ${R}_{{eq}}=\,5.6\times {10}^{3}$ mmHg.s/mL. This is obtained using Murray’s Law, where the resistance *R*_*i*_ at a face *i* is given as *R*_*i*_ = *CA*_*i*_^−1.5^. *A*_*i*_ is the cross-sectional area of the outlet and *C* is a constant calculated such that ${R}_{{eq}}^{-1}={\sum }_{i=1}^{{N}_{{faces}}}{R}_{i}^{-1}$. Note that varying this resistance or the aortic wall velocity had a minimal effect on the reported results. Simulations were performed on the idealized geometries displayed in Supplementary Fig. 18 to capture the effects of the misplaced LCO present in the *Sox17*^*eKO*^ relative to the normally placed LCO in the control. Furthermore, simulations were performed on non-idealized geometries extracted from clinical images. However, these images had a high level of uncertainty and variability due to their limited resolution, with phenomena such as artificial narrowing occurring in coronary branches. As such, the idealized versions provide a better avenue for comparing the effects, at least qualitatively, of the placement of the LCO, as these types of factors can be more carefully controlled. In addition to these two sets of cases, simulations with Newtonian viscosities were performed on the idealized geometries with a constant viscosity of 4 × 10^−3 ^Pa.s. The results from these simulations are similar to those from the non-Newtonian simulations, showing a drop in WSS of similar magnitude between the control and *Sox17*^*eKO*^ cases. The Newtonian results showed somewhat higher values of WSS overall because the regions of the flow where the WSS is highest necessarily coincide with the regions where the shear rate is highest. This causes a reduction in the viscosity in these regions, and thus, the WSS.

### RNA in situ hybridization (ISH)

RNA ISH was performed using the RNAscope kit from Advanced Cell Diagnostics (ACD) according to the manufacturer protocol. Briefly, embryonic hearts were freshly isolated in PBS, fixed in 4% PFA at 4 °C overnight, washed in PBS, soaked in 15% and 30% sucrose sequentially and embedded in OCT compounds with orientation for transverse sections. The hearts were then sectioned at 8 μm, the sections were mounted on positive charged slides and stored at −80 °C. RNA probe for mouse *Pdgfb* or human *PDGFB* was purchased from ACD and the RNAscope 2.0 assay kit was used according to the manufacturer’s instruction. Note that the RNA probe in human *PDGFB* does not recognize the mouse *Pdgfb*. After RNAScope probe ISH, an IB4 antibody was staining as above described. Images were taken by a Leica SP5 confocal microscope and imaging quantifications were carried out blindly using Image J.

### Chromatin immunoprecipitation quantitative PCR (ChIP-qPCR)

Immortalized MCEC (CEDARLANE, Cat #: CLU510), prepared from microvascular neonatal MCEC by transfection with lentiviral vectors carrying SV40 T antigen and human telomerase, were used to do ChIP. Briefly, about 1 × 10^7^ Cells were homogenized with 1% formaldehyde and incubated at RT for 15 min with rotation. Glycine was then added to quench the cross-link reaction. After centrifuge (2000 *g* × 5 min), the pellets were resuspended in nuclei lysis buffer (1% SDS, 10 mM EDTA, 50 mM Tris, pH 8.1) for sonication to shear chromatin with Bioruptor (Diagenode) (30 s on, 30 s off, power setting H, 30 min, twice) to generate the fragments at the range of 100–300 bp. Sonicated chromatin was pre-cleaned by ChIP-Grade Protein G magnetic beads (9006, Cell Signaling Technologies) and immunoprecipitated with anti-SOX17 antibody overnight. ChIP-Grade Protein G magnetic beads were added to collect the immunoprecipitated complex and washed once with low salt wash buffer (0.1% SDS, 1% Triton X-100, 2 mM EDTA, 20 mM Tris-HCl, pH 8.1, 150 mM NaCl), high salt wash buffer (0.1% SDS, 1% Triton X-100, 2 mM EDTA, 20 mM Tris-HCl, pH 8.1, 500 mM NaCl), LiCl wash buffer (0.25 M LiCl, 1% IGEPAL-CA630, 1% deoxycholic acid (sodium salt), 1 mM EDTA, 10 mM Tris, pH 8.1) and twice with TE buffer (10 mM Tris-HCl, 1 mM EDTA, pH 8.0). Quantitative PCR was then performed with primers as follows:

Mouse *Pdgfb* enhancer 1-F, AGACAACTTGGCATGGGTCAGG

Mouse *Pdgfb* enhancer 1-R, ATCAGACAGACTAAATTTAGAGTG

Mouse *Pdgfb* enhancer 2-F, AAACAGCAAGAAAGCTAAATTTGG

Mouse *Pdgfb* enhancer 2-R, TGGTAAGGCAGGCAGGAGGG

### Luciferase reporter gene assay

The mouse Pdgfb distal enhancer and gene body enhancer with or without the SOX17 binding site was PCR cloned into the PGL3 luciferase reporter vector at Sal1 and BamH1. All constructs were confirmed by Sanger sequencing. Lipofectamine LTX and PLUS Reagents (Life Technologies) were used for transfection of the PGL3 luciferase reporter vector into the immortalized MCECs. The Renilla luciferase vector was co-transfected and used for normalizing transfection efficiency using the dual-luciferase reporter assay kit (Promega, E1980) according to the manufacturer’s instructions. All data were normalized by Renilla luciferase luminescence derived from the co-transfected pRL-SV40 vector (Promega, E2231).

### RNA-sequencing and quantitative real time reverse transcriptase reaction (RT-qPCR)

Total RNAs were isolated from the cardiac OFT of E12.5 hearts using Trizol (Invitrogen). Total RNAs were checked for quality and quantity on an Agilent Bioanalyzer 2100, with an RNA Integrity Number (RIN) of at least 8.0. Libraries were prepared according to the Illumina TrueSeq RNA library method using the “TrueSeq RNA Sample preparation guide” (Illumina Technologies). The fragment distribution of libraries was examined on an Agilent High Sensitivity DNA BioAnalyzer chip, quantified using real-time PCR, and used to prepare clusters using a One-Touch 2 device. These clusters were then sequenced on Illumina HiSeq 2500, to generate 100 bp paired reads. With two biological replicates for control and Sox17^eKO^ samples, we obtained on average ~30 million pairs of reads per sample for RNA-seq data analysis. RT-qPCR validation of RNA-sequencing results was performed using the Power SYBR Green PCR Master Mix (ABI). Gene specific primers were listed in Supplementary material, Supplementary Table 3. The relative level of gene expression was normalized to an internal control using Gapdh and calculated using the 2^−ΔΔCT^method. Biological repeats were performed using three individual OFT samples from each genotype, technical triplicates were carried out for each sample for PCR.

### RNA-sequencing data and bioinformatics analysis

Adapter and low quality bases in RNA-seq reads were trimmed by Trim Galore. Trimmed reads were aligned to the mouse genome (mm10) using Tophat (version 2.0.13)^102^, with default parameters. Based on GENCODE database (vM6), the number of RNA-seq fragments mapped to each gene was determined by HTSeq^103^. DESeq2^104^ was then used to determine differentially expressed genes (DEGs) from the gene-level read counts at adjusted *p* values (padj) < 0.1 (Supplementary Data 1). Function enrichment analysis of the DEGs was conducted for protein-coding genes by Toppgene^105^ and Reactome^106^. The RNA-seq data has been deposited to the GEO database (GSE129564).

### Reanalysis of ChIP-Seq data and comparison with differential expressed genes

SOX17 ChIP-seq reads in mouse embryonic stem cells overexpressing SOX17^53^ were downloaded from the GEO (GSE43275) and aligned to the mouse genome (mm10) by bowtie (version 2.3.3.1)^107^. The alignments were converted to bam files by samtools^108^. Uniquely mapped reads were kept for peak calling by MACS2^109^, using input as controls. Genes with at least one peak from 50 kb upstream of the transcription start sites to 50 kb downstream of transcription terminus were defined as SOX17-binding genes. These SOX17-binding genes were compared to the DEGs from our RNA-seq analysis to classify DEGs into two categories: DEGs with SOX17-binding sites and those without.

### Statistics and reproducibility

Student’s *t* test (two-tailed) or one-way ANOVA analysis followed by Tukey’s test was used for statistical difference between or among groups, assuming unequal variance among samples. The data were plotted as column with scattered points using GraphPad Prism 8.3.0. Normality was assumed and variance was compared between groups. All experiments were reproduced at least three times using biological different samples, except for the transcriptomic analyses which were carried out twice with independent samples of pooled cardiac OFT from 10 hearts of E12.5 control and *Sox17*^*eKO*^ embryos, respectively. The investigators were not blinded to the group allocation and the outcome assessment during experiments, unless stated otherwise. Numerical data were presented as mean ± SD and *p* value of <0.05 was considered as significant.

### Reporting summary

Further information on research design is available in the Nature Research Reporting Summary linked to this article.

## Supplementary information

Supplementary Information

Reporting Summary

Description of Additional Supplementary Files

Supplementary Data 1

## Data Availability

The data supporting the findings from this study are available within the paper and its supplementary information. The RNA-seq data has been deposited to the GEO database (GSE129564). Source data are provided with this paper.

## Source data

Source Data
