# Supplementary material for: A SOX17-PDGFB signaling axis regulates aortic root development
Source: Nat Commun. 2022 Jul 13;13:4065. doi: 10.1038/s41467-022-31815-1 (PMC9279414; doi:10.1038/s41467-022-31815-1)
Supplement: Supplementary file 1 — Supplementary Information [file 41467_2022_31815_MOESM1_ESM.pdf]

Supplementary Information

Supplementary Figs and Figure Legends

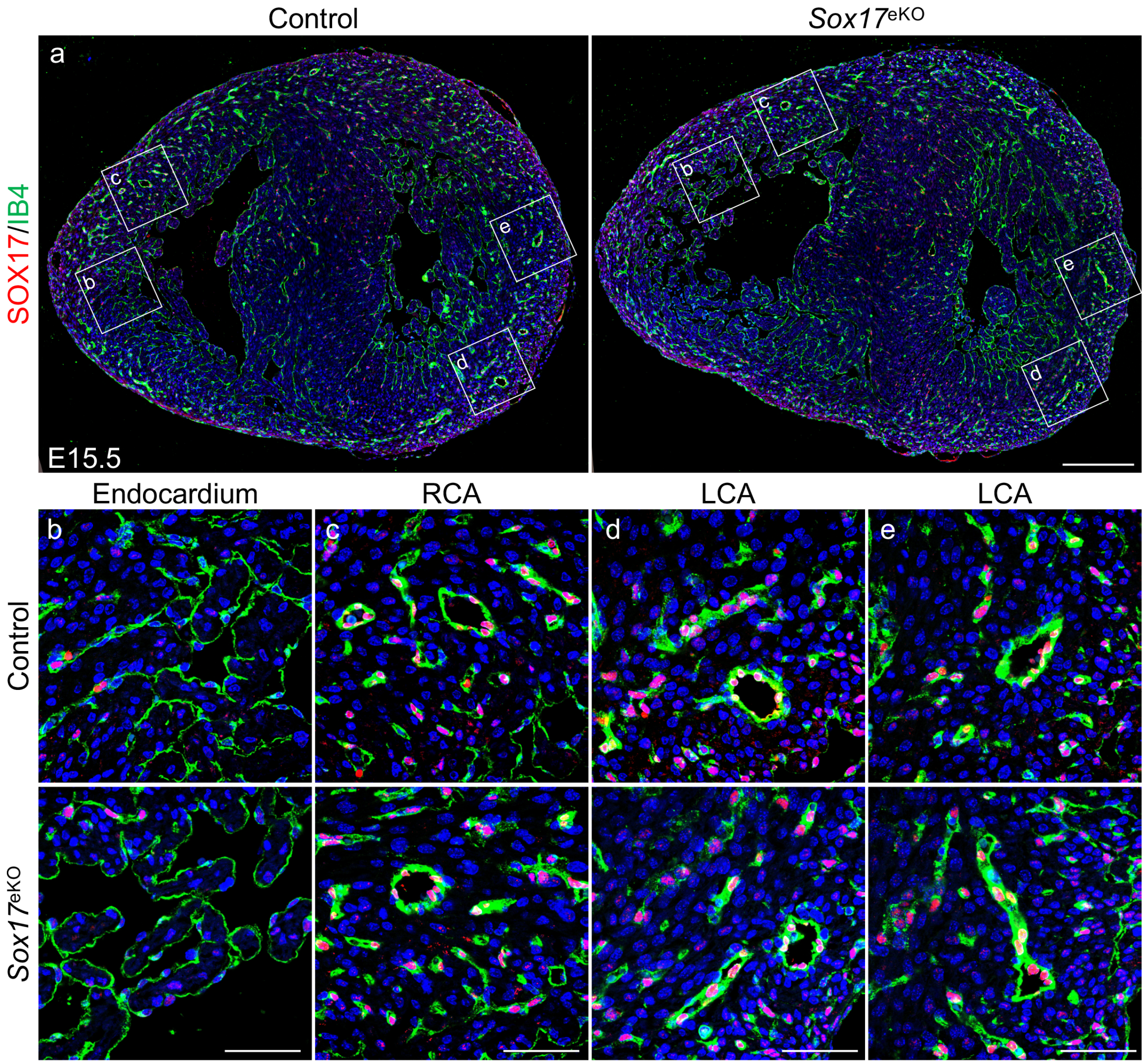

Supplementary Fig 1. The SOX17 expression pattern in the endocardium and the coronary arteries was not altered in E15.5 *Sox17*$^{\mathbf{eKO}}$ hearts. a Co-IF of SOX17 (red) and IB4 (green) (n=5/group) shows similar SOX17 expression (red) in the ventricle of E15.5 control and *Sox17*^{eKO} hearts. **b**, **c**, **d**, **e** Boxed regions in **a** showed the endocardium region (**b**), right coronary artery (RCA) (**c**), and left coronary artery (LCA) (**d** and **e**). Noted that SOX17 is not expressed in the endocardium in both control and *Sox17*^{eKO} hearts. The SOX17 expression in the coronary arteries is not altered in E15.5 *Sox17*^{eKO} hearts. Scale bar: 200um in **a**; 50um in **b**, **c**, **d** and **e**.

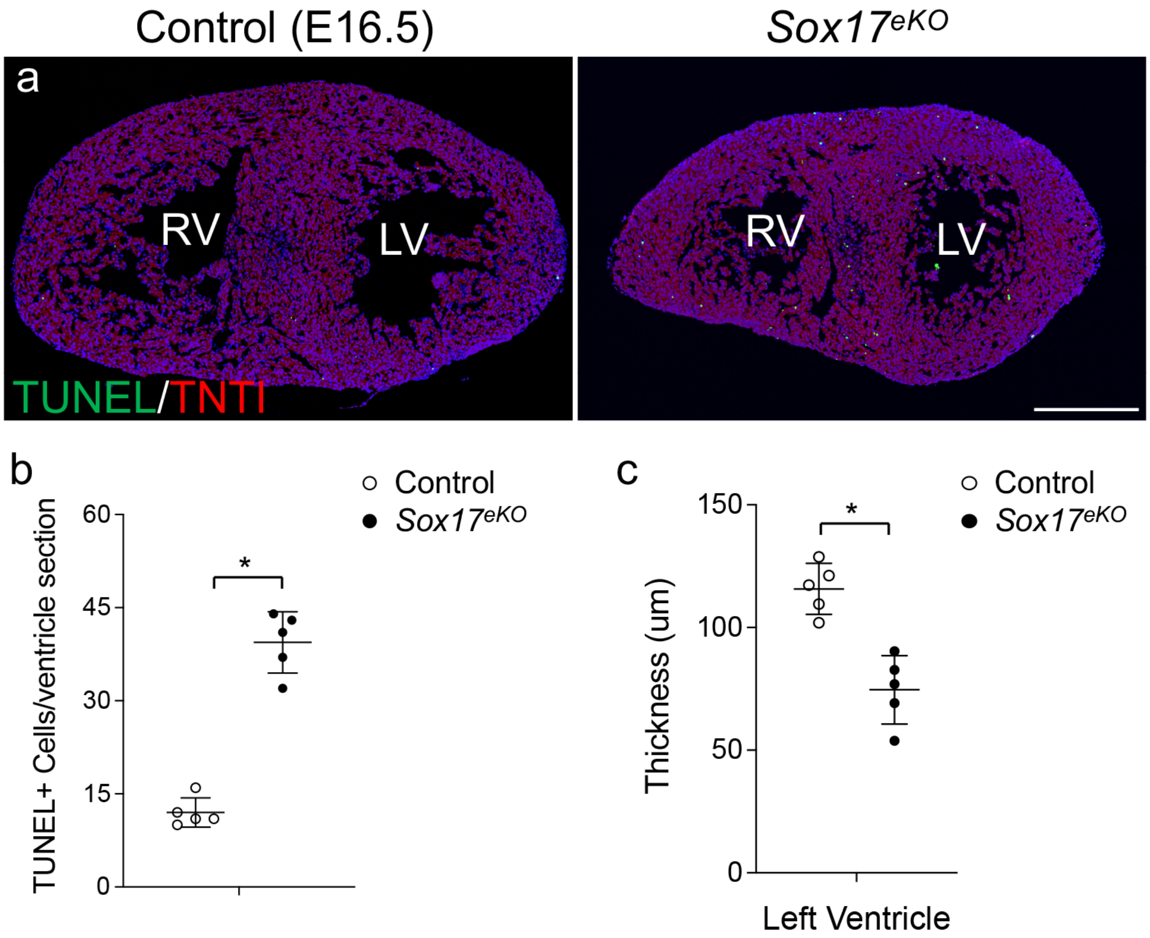

Supplementary Fig 2. The *Sox17* deletion results in increased apoptosis in the ventricular wall. a, **b** Quantitative TUNEL labeling (green) and myocardial staining (TNTI, red) shows increased apoptotic cells in the left ventricular wall of E16.5 $Sox17^{eKO}$ hearts. n=5/group, mean ± SD, unpaired two-tailed *t*-test, *p*=0.000004, **p*<0.05. **c** Quantitative analysis showed significant reduced thickness of the left ventricular free wall of E16.5 $Sox17^{eKO}$ hearts. n=5/group, mean ± SD, unpaired two-tailed *t*-test, *p*=0.0007, **p*<0.05. Source data are provided as a Source Data file. Scale bars: 100 μm.

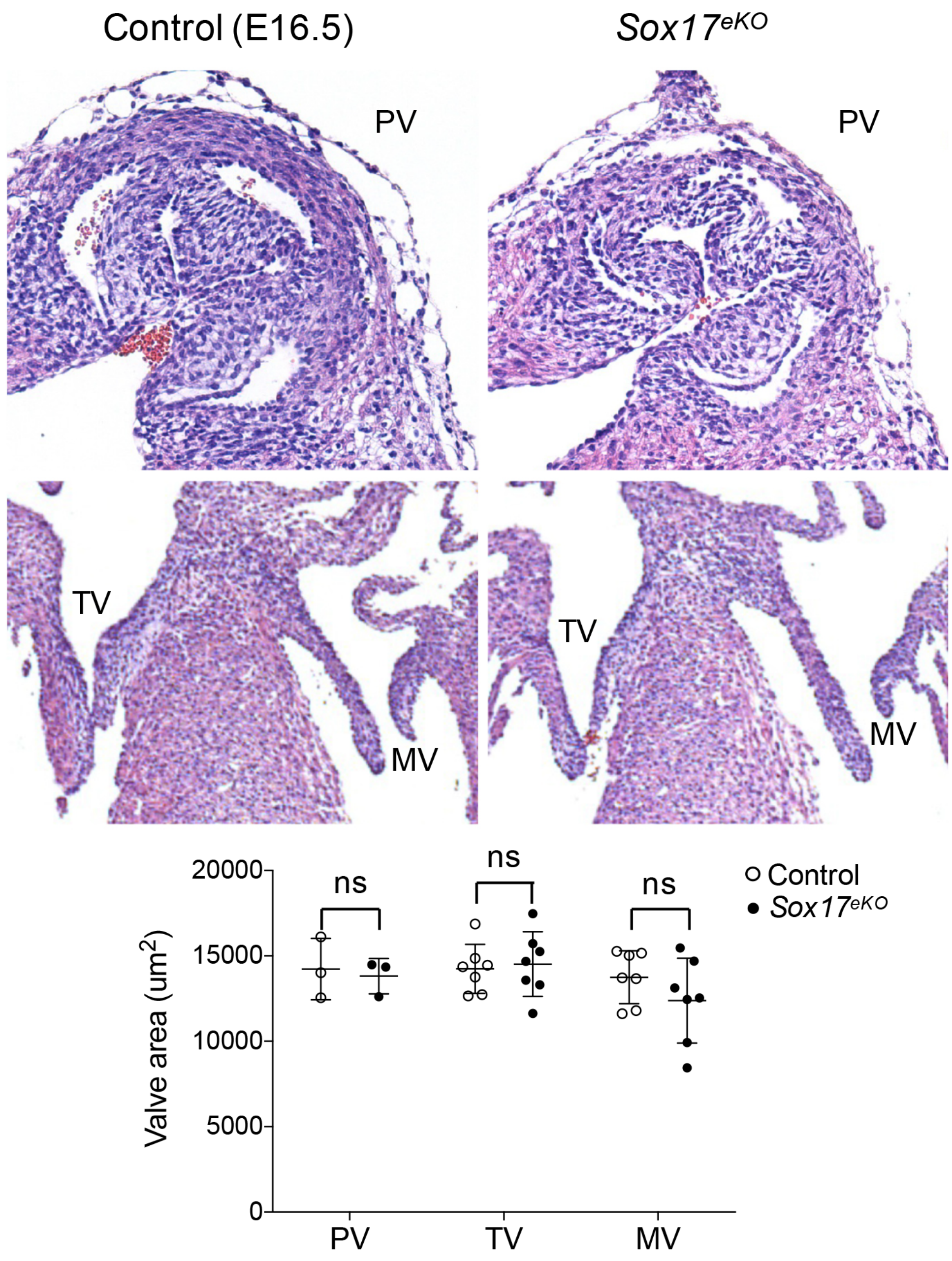

Supplementary Fig 3. *Sox17* deletion does not affect the development of pulmonary, mitral and tricuspid valve. H&E stained sections of pulmonary valve (PV, n=3/group), mitral valve (MV, n=7/group) and tricuspid valve (TV, n=7/group) of E16.5 hearts and quantification of valve area indicate that their development is not affected by the *Sox17* deletion. (mean ± SD, unpaired two-tailed *t*-test, $p<0.05$ as significant). Source data are provided as a Source Data file.

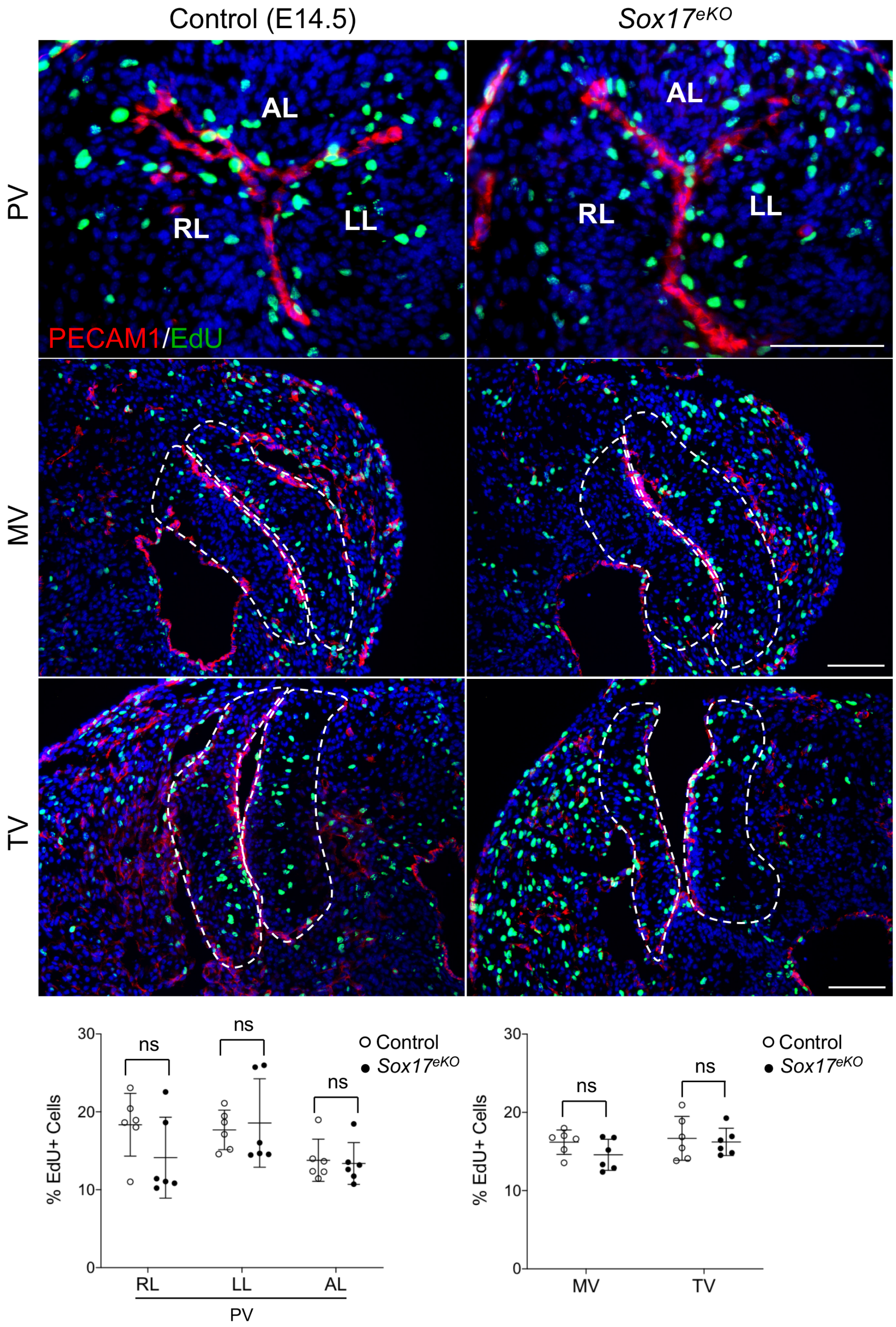

Supplementary Fig 4. *Sox17* deletion does not affect cell proliferation in pulmonary, mitral and tricuspid valves. EdU (green) labeling assay shows cell proliferation in PV, TV, and MV, in the outlined region, of E14.5 control and *Sox17*^{eKO} hearts. PECAM1 staining (red) demarks valve structure. AL/RL/LL, anterior/right/left leaflet of pulmonary valve. (n=6/group, mean ± SD, unpaired two-tailed *t*-test, p<0.05 as significant). Source data are provided as a Source Data file. Scale bar: 100 μm.

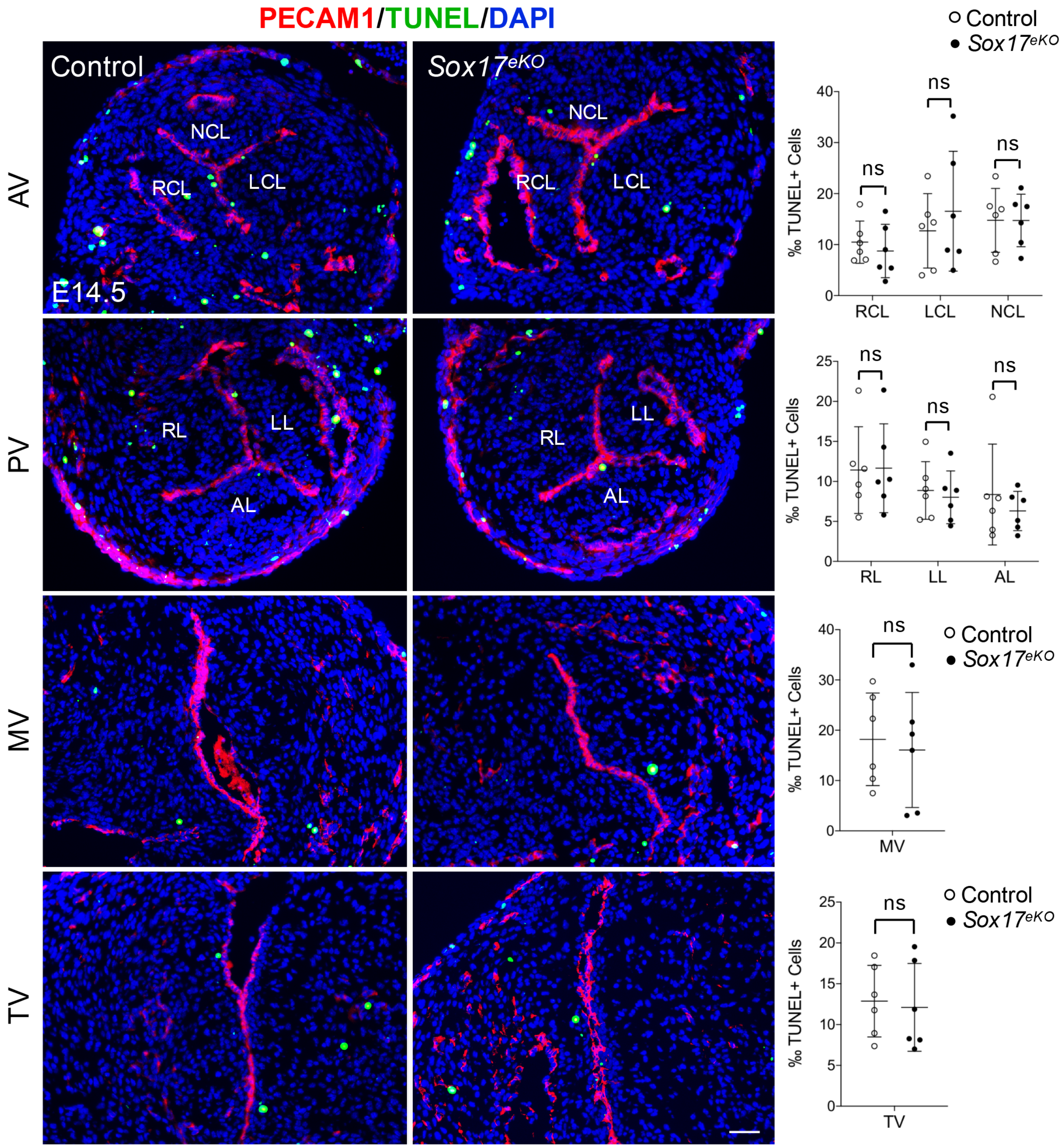

Supplementary Fig 5. *Sox17* deletion does not affect cell apoptosis in developing heart valves. TUNEL labeling (green) assay and quantification data shows the percentage of apoptotic cells in aortic, pulmonary, mitral and tricuspid valve (AV, PV, TV and MV) between control and *Sox17*^{eKO} hearts was similar. PECAM1 staining (red) demarks valve structure. NCL/RCL/LCL, non-/right/left coronary leaflet of aortic valve; RL/LL, anterior/right/left leaflet of pulmonary valve. (n=6/group, mean ± SD, unpaired two-tailed *t*-test, $p<0.05$ as significant). Source data are provided as a Source Data file. Scale bars: 100 µm.

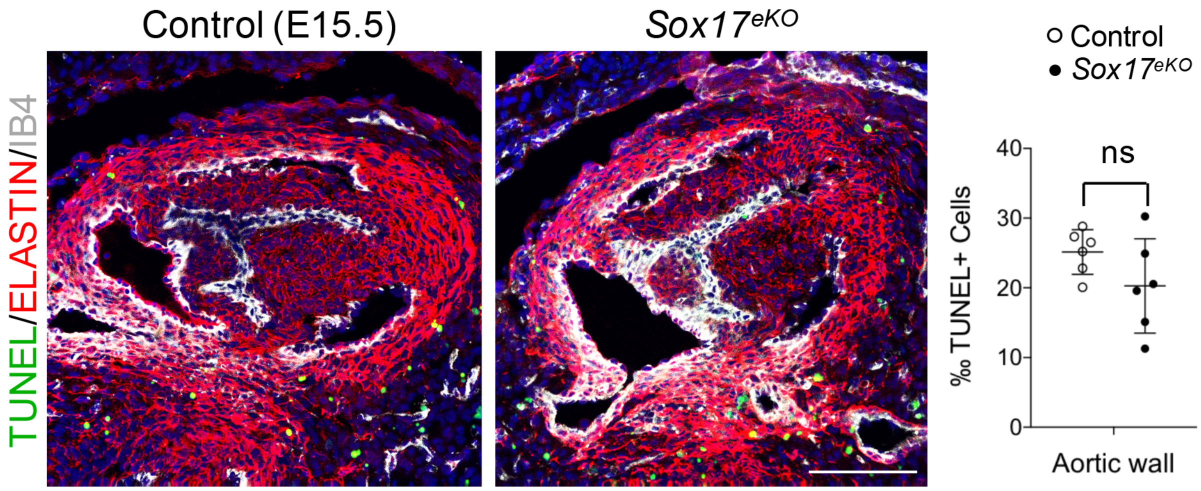

Supplementary Fig 6. *Sox17* deletion does not affect apoptosis of VSMC of aortic wall. TUNEL labelling (green) assay shows the percentage of apoptotic cells (red) in the aortic wall was not altered by the *Sox17* deletion. (n=6/group, mean ± SD, unpaired two-tailed *t*-test, $p<0.05$ as significant). Source data are provided as a Source Data file. Scale bar: 100µm.

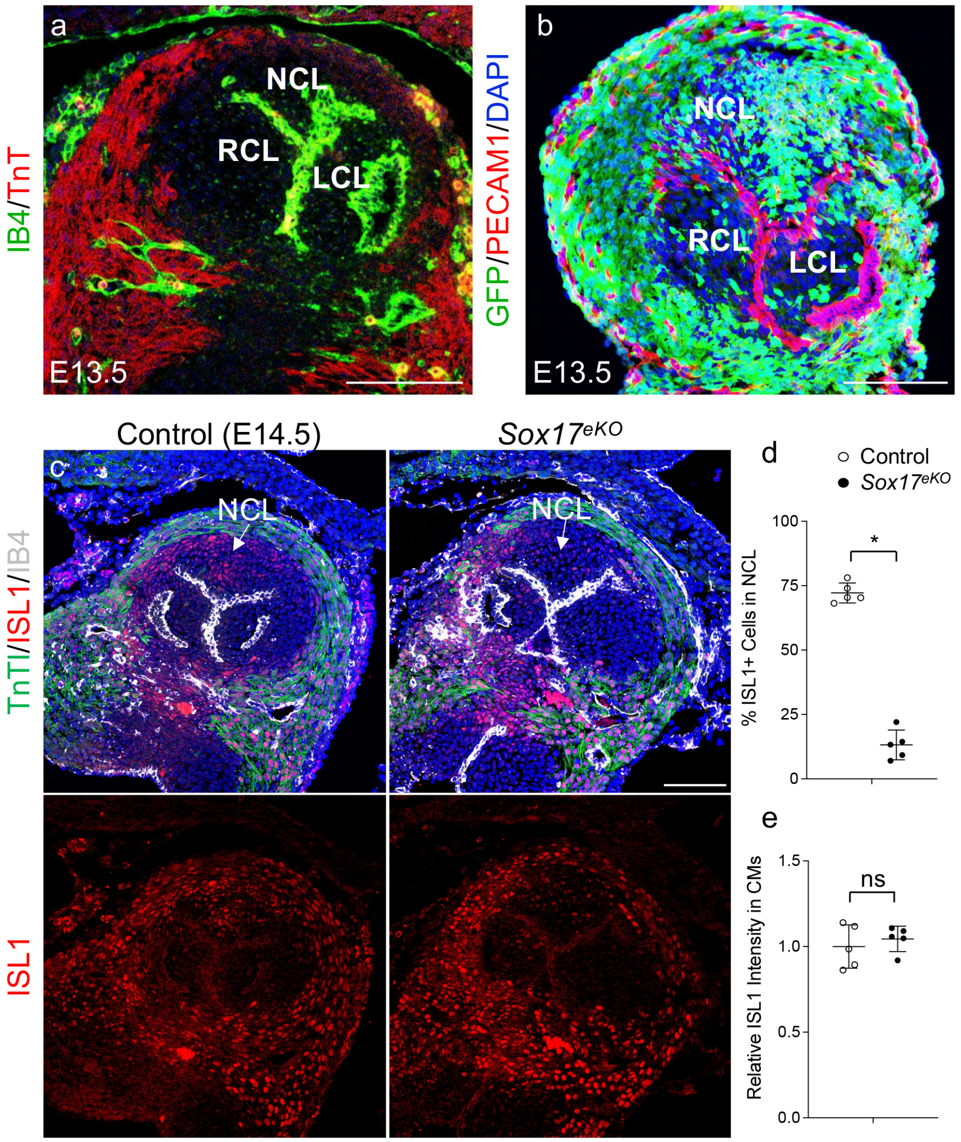

Supplementary Fig 7. a IF (n=6/group) shows the cardiac TnT protein (red) is not expressed in the mesenchyme of aortic valve leaflets and the muscular wall of aortic sinuses related to NCL and LCL in E13.5 heart. IB4 IF (green) demarks the aortic root (valve and sinus) endothelium. **b** Lineage tracing (n=4/group) using the TnT-Cre mediated expression of GFP (green) shows a significant contribution of cardiomyocyte lineage to the NCL mesenchyme and the muscular wall of aortic sinuses related to NCL and LCL. PECAM1 IF (red) labels the aortic root (valve and sinus) endothelium. These data support the NCL mesenchyme and aortic root VSMCs share a SHF cardiomyocyte progenitor origin. **c**, **d**, **e** Representative IF images and quantitative analysis of E14.5 control and $Sox17^{eKO}$ hearts show decreased percentage of ISL1 (red) in the NCL mesenchyme (arrow) while the relative ISL1 expression level in the cardiomyocytes (marked by TnTI, green) was not altered. (n=5/group, mean ± SD, unpaired two-tailed *t*-test, p=0.00000006 for **d**, *p<0.05). Source data are provided as a Source Data file. Scale bars: 100µm.

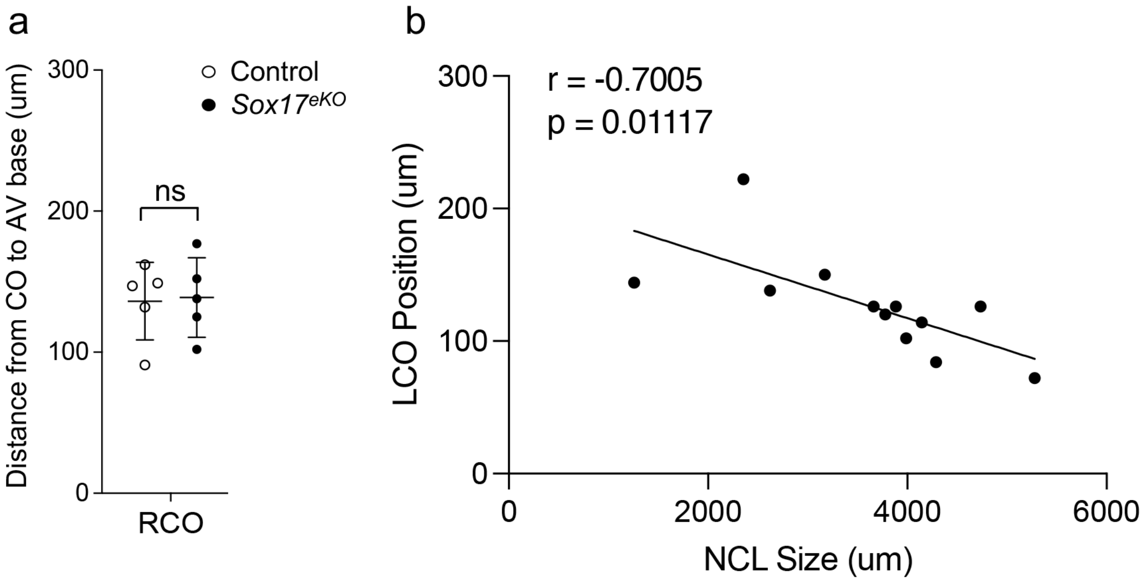

Supplementary Fig 8. a Quantitative analysis of the position of RCO shows no difference between E16.5 control and *Sox17*^{eKO} hearts. (n=7 for control, n=5 for *Sox17*^{eKO}, mean ± SD, unpaired two-tailed *t*-test). **b** Pearson correlation analysis between the NCL size with the LCO position shows they are significantly negatively correlated. n=7 for control, n=5 for *Sox17*^{eKO}, two-tailed, correlation coefficient *r* and *p* value are indicated in the graph. Source data are provided as a Source Data file.

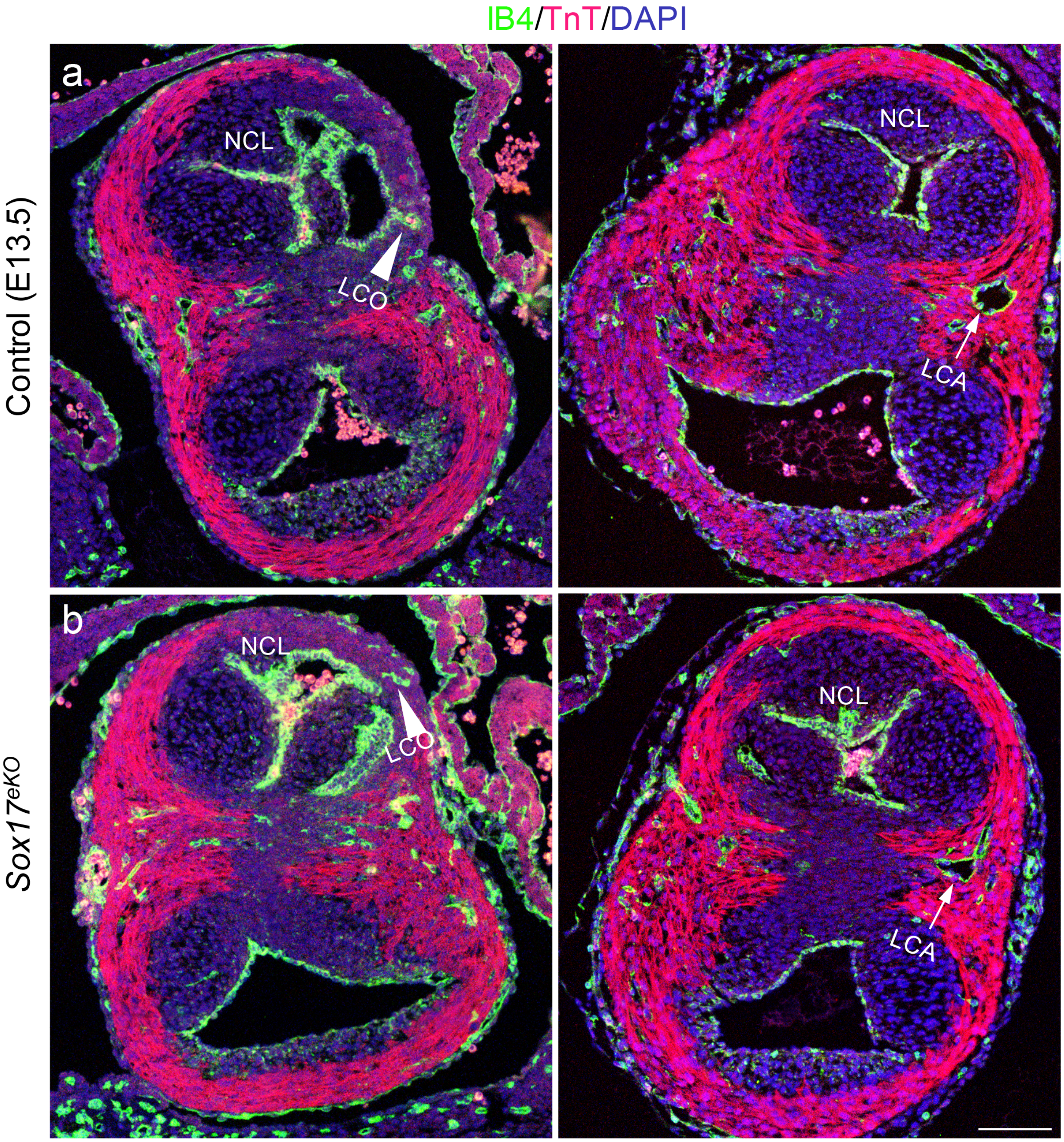

Supplementary Fig 9. a, **b** IF (n=5/group) for TnT (red) and IB4 (green) shows the nascent left coronary ostium (LCO, arrowhead) forming in the anterior part of left aortic sinus (LAS) wall negative for TnT expression in E13.5 control heart (**a**). In E13.5 *Sox17*^{eKO} heart, the primitive LCO (arrowhead) is seen in more posterior part of LAS (**b**). Arrow indicates the primitive left coronary artery (LCA) which develops in comparable location in control and *Sox17*^{eKO} heart. Scale bars: 100µm.

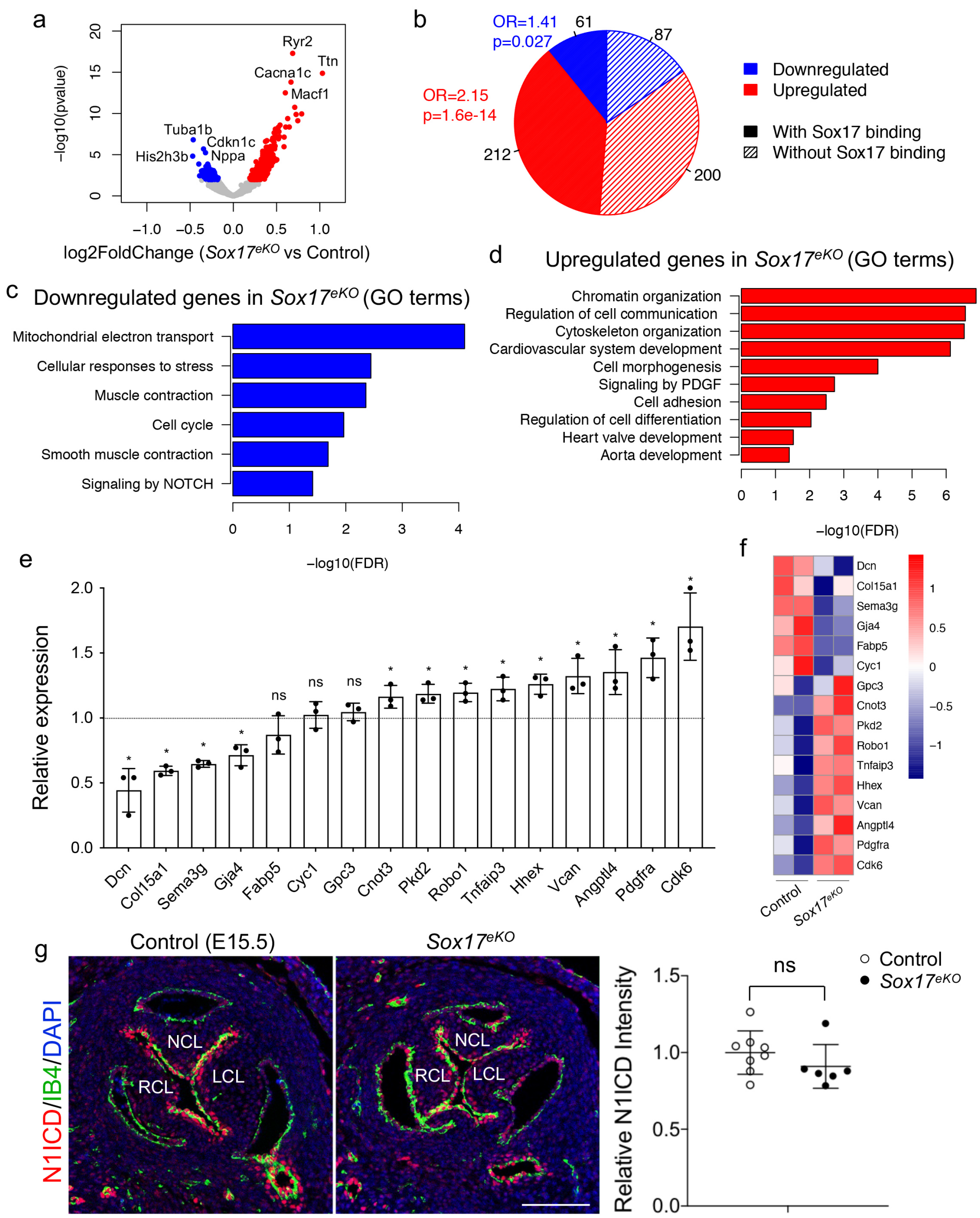

Supplementary Fig. 10. SOX17 regulates genes essential for aortic root maturation. a-d RNAseq analysis of E12.5 OFTs. **a** Volcano plot shows genes with increased (red) or decreased (blue) expression in E12.5 *Sox17*^{eKO} OFTs. **b** Pie chart shows differential expressed genes (DEGs) in E12.5 *Sox17*^{eKO} OFTs are enriched for SOX17 binding sites described in mouse embryonic stem cells (ESCs) [1]. **c**, **d** Gene Ontology enrichment analysis for DEGs in the OFTs of E12.5 *Sox17*^{eKO} hearts shows the SOX17-dependent genes involved in NOTCH signaling, PDGF signaling and cardiovascular development as three of top functional categories. **e** RT-qPCR confirms the altered expression of genes selected from the DEGs. (n=3/group, mean ± SD, unpaired two-tailed *t*-test, *p*=0.008 for Dcn, *p*=0.0008 for Col15a1, *p*=0.04 for Sema3g, *p*=0.04 for Gja4, *p*=0.03 for Cnot3, *p*=0.01 for Pkd2, *p*=0.02 for Robo1, *p*=0.01 for Tnfaip3, *p*=0.02 for Hhex, *p*=0.02 for Vcan, *p*=0.02 for Angptl4, *p*=0.02 for Pdgfra, *p*=0.02 for Cdk6, **p*<0.05). **f** Heatmap showing the expression in RNA-seq of the verified DEGs in **e**. **g** IF images and quantitative analysis of E15.5 control and *Sox17*^{eKO} hearts show comparable N1ICD expression (red) in aortic valve endothelium (n=8 for control, n=6 for *Sox17*^{eKO}. mean ± SD, unpaired two-tailed *t*-test, *p*=0.26). Source data are provided as a Source Data file. Scale bars: 100μm.

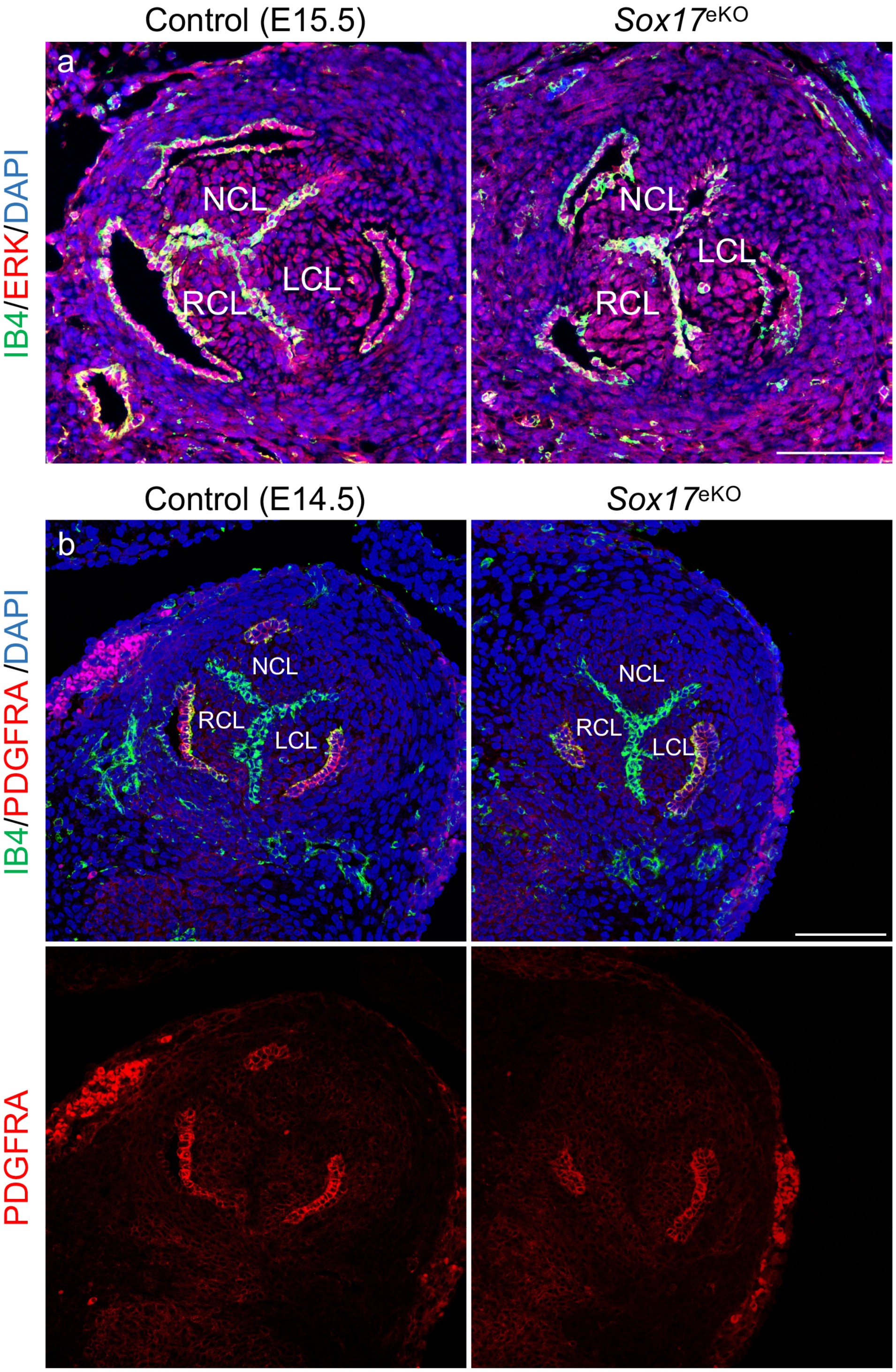

Supplementary Fig. 11. Total ERK and PDGFRA expression pattern in the aortic root. a Representative total ERK immunostaining (red) images (n=3/group) show similar expression pattern in the aortic valve region between E15.5 control and *Sox17*^{eKO} hearts. **b** Representative PDGFRA immunostaining (red) images (n=5/group) show PDGFRA is expressed in the aortic valve endothelium on the sinus side and aortic wall adventitia, and the expression is not altered in the aortic valve region of E14.5 *Sox17*^{eKO} hearts. Scale bars: 100μm.

```
                       SMAD    NF-1                                 EIIaE-A
Mouse           TATGCCCAGACAACTTGGCATGGGTCAGGATCTGCCATTTAGCAAGCAGCCTGCCCTCTG
Rat             TGTACCCAGACAACTTGGCATGGATCAGGATCTGTCATTTAGCAAGCGGCCTGCCCTCTG
Rhesus          CATGCCCAGACAGCTTGGCATGGCTCAG-ACCCAGCATTCAACGGGCAGCCTGCCCTCTG
Human           CATGCCCAGACAGCTTGGCATGGCTCAG-ACCCAGCATTCAACGGGCAGCCTGCCCTCTG
                 .*.********.********** **** * * . **** *.*..**.************

                 SP3        SOX17         ELK-1 SOX17
Mouse           GCCCCTCATGAACAATGGGGACTCCAGGAATATTGTCTA-CCCGGACTGAATCAAACACT
Rat             GCCCCTCAGGAACAATAGGGACTCCAGGAATATTGTCTA-CCCGGACTGAATCAAACACT
Rhesus          GCCCCTCACAAACAATGGGGACTCCAGGAATATTGTCCGGCCCGGGCTGAATCTAATGCT
Human           GCCCCTCACAAACAATGGGGACTCCAGGAATATTGTCCCGCCCGGGCTGAATCAAACGCT
                ******** .******.********************   *****.*******:** .**

                             SMAD     SP1      ETS-a SP3
Mouse           CTAAATTTAGTCTGTCTGATGCGCCCTCTGTTTCCTGCCCCTGTTGCCCTCCTTTCAAGG
Rat             CTAAATTTAGTCTGTCTGATGCGCCCTCTGCTTCCTGCCCCTGTTGCCCTCCTTTCAAGG
Rhesus          CTAAATTTAGTCCCTCTGATGCGTCCTCCGTTTCCTGCCCCTGCTGCCCTCCTTTCAAGG
Human           CTAAATTTAGTCTCTCTGATGTGTCCTCCGTTTCCTGCCCCTGCTGCCCTCCTTTCAAGG
                ************  ******* * **** * ************ ****************

                          SPE    ETS-b
Mouse           CGATGAGGTCACCCCGGAACTGCATGCCTTGGCAGCCAGACCCAGGGGGTTTCCAGGTCT
Rat             CGATGAGGTCACCCCGGAACTGCATGTCTTGGCAACCAGACCCAGGGGGTTTCCAGGTCT
Rhesus          CGATGAGGTCACCCCGGAACCGCCTGCCCCAGCAGCCAGACCCAGGGGGTTTCCAGGCCC
Human           CGATGAGGTCACCCCGGAACTGCCTGCCCCAGCAGCCAGACCAAGGGGGTTTCCAGGCCC
                ******************** **.** *  .***.*******.************** *
```

Supplementary Fig. 12. Multispecies alignment of the conserved region of the *Pdgfb* enhancer 1 using ClustalW. Red sequences are potential *Sox17* binding motifs and gray sequences are the putative binding motifs for other transcription factors identified in silico.

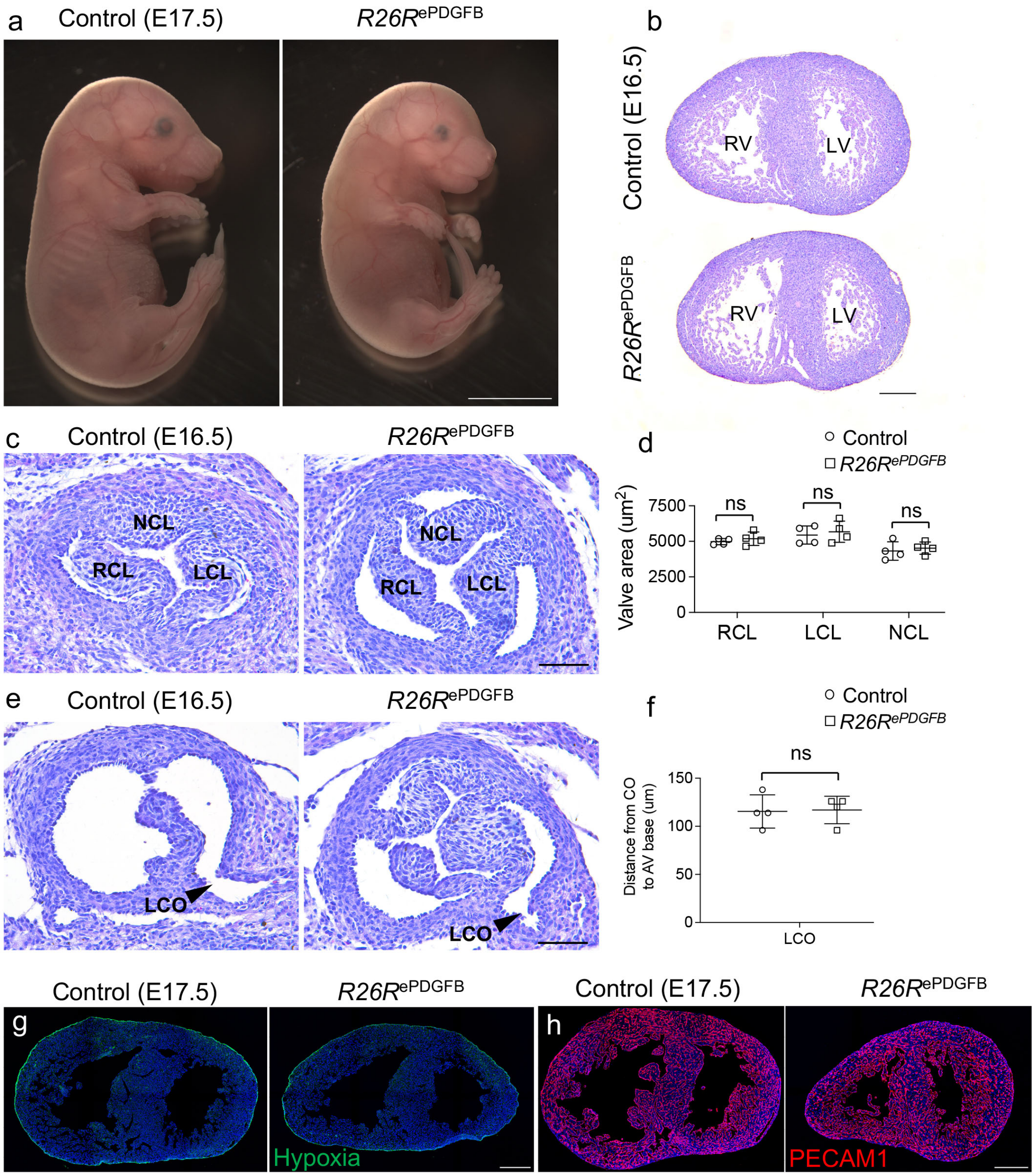

Supplementary Fig. 13. PDGFB over-expression hearts develop normally. a Representative image (n=3/group) of E17.5 control and *R26R*^{ePDGFB} hearts show that the *R26R*^{ePDGFB} embryos are somewhat underdeveloped. **b**, **c** H&E staining (n=4/group) showing normal hearts and NCL, respectively, in all 4 E16.5 *R26R*^{ePDGFB} embryos examined. LV/RV: left and right ventricle. **d** Quantitative analysis shows comparable NCL area in E16.5 *R26R*^{ePDGFB} and control hearts. n=4/group, mean ± SD, unpaired two-tailed *t*-test, ns, no significance. **e** Representative H&E stained images of E16.5 control and *R26R*^{ePDGFB} heart shows that LCO is similar positioned in E16.5 *R26R*^{ePDGFB} hearts compared to the controls. Arrowheads indicate the LCO. **f** Quantitative analysis shows no difference in the LCO position related to aortic valve base between *R26R*^{ePDGFB} hearts and controls. n=4/group, mean ± SD, unpaired two-tailed *t*-test, ns, no significance. **g, h** Representative hypoxia probe staining (green in **g**) and PECAM1 staining (red in **h**) (n=3/group) show the similar staining pattern and intensity between E17.5 control and *R26R*^{ePDGFB} hearts. Source data are provided as a Source Data file. Scale bars: 5mm in a; 200 µm in b, g, h; 100 µm in c, e.

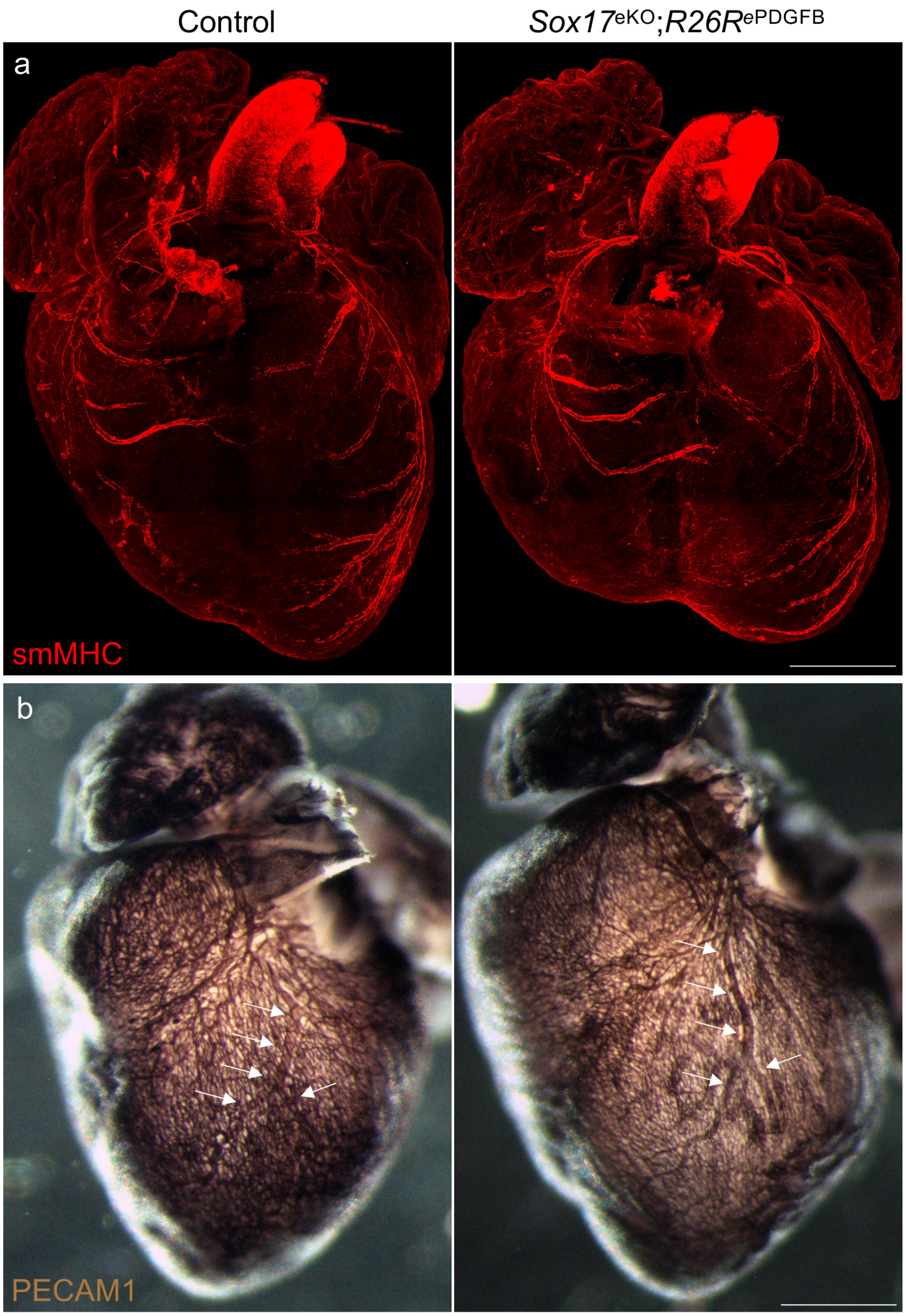

Supplementary Fig. 14. PDGFB re-expression rescues the coronary artery defects. a Representative whole heart smMHC IF (red) images of E16.5 control and *Sox17^{eKO};R26R^{ePDGFB}* hearts (n=3/group) showing normal coronary artery development. **b** Representative images of PECAM1 stained (gray) E16.5 control and *Sox17^{eKO};R26R^{ePDGFB}* hearts (n=3/group) show normal coronary artery development. Arrows indicate left coronary artery. Scale bars: 500 µm.

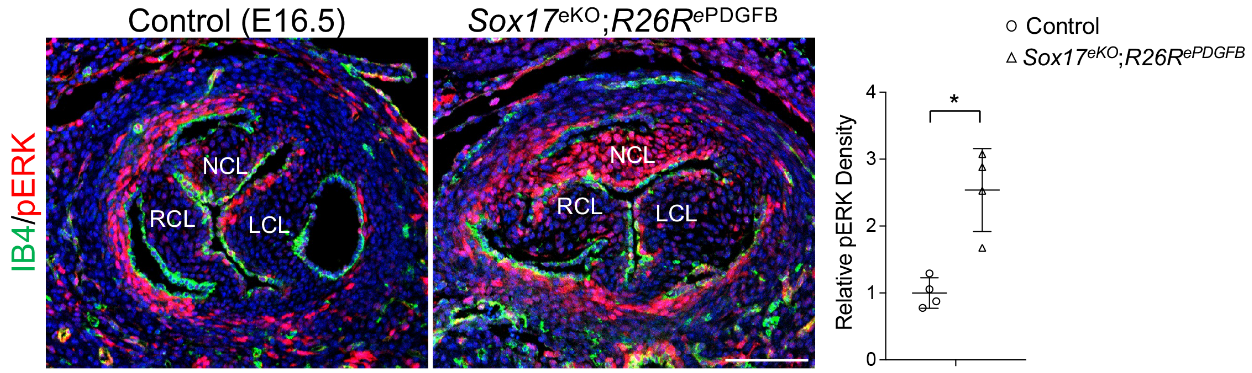

Supplementary Fig. 15. Representative phosphorylated ERK (pERK) (red) IF images show continuing highly pERK expression in the NCL mesenchyme of E16.5 *Sox17^{eKO};R26R^{ePDGFB}* hearts comparing to control hearts. n=4/group, mean ± SD, unpaired two-tailed *t*-test, *p*=0.003, **p*<0.05. Source data are provided as a Source Data file. Scale bars: 100 μm.

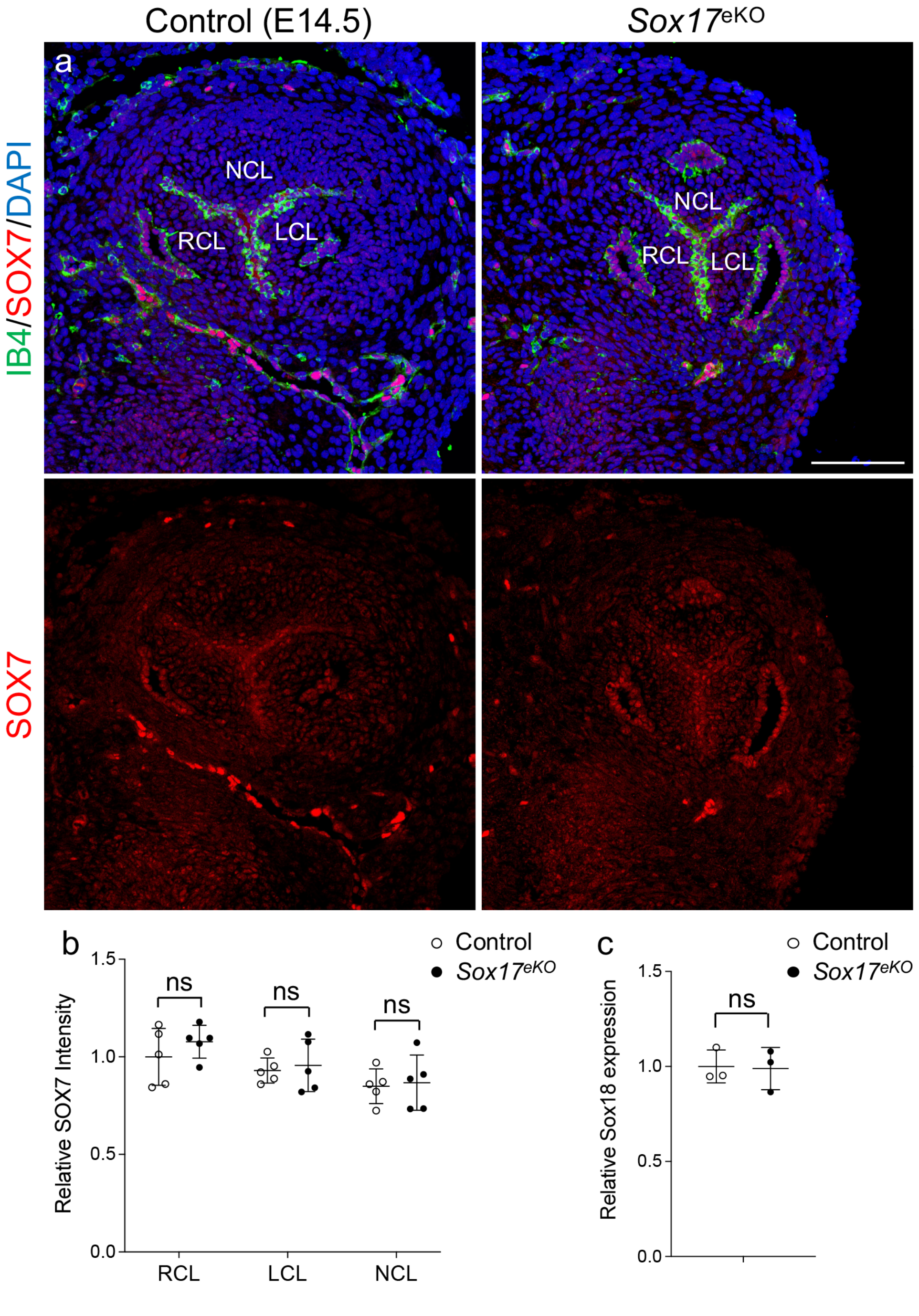

Supplementary Fig. 16. ***Sox17*** **deletion does not affect the expression of SOX7 and** ***Sox18*****. a**, **b** Representative images and quantitative analysis of E14.5 control and *Sox17^{eKO}* hearts show comparable SOX7 expression (red) in the aortic region. n=5/group, mean ± SD, unpaired two-tailed *t*-test, ns, no significance. **c** RT-qPCR analysis of E12.5 OFTs shows the *Sox18* expression is not altered in the *Sox17^{eKO}* hearts. n=3/group, mean ± SD, unpaired two-tailed *t*-test, ns, no significance. Source data are provided as a Source Data file. Scale bars: 100 µm.

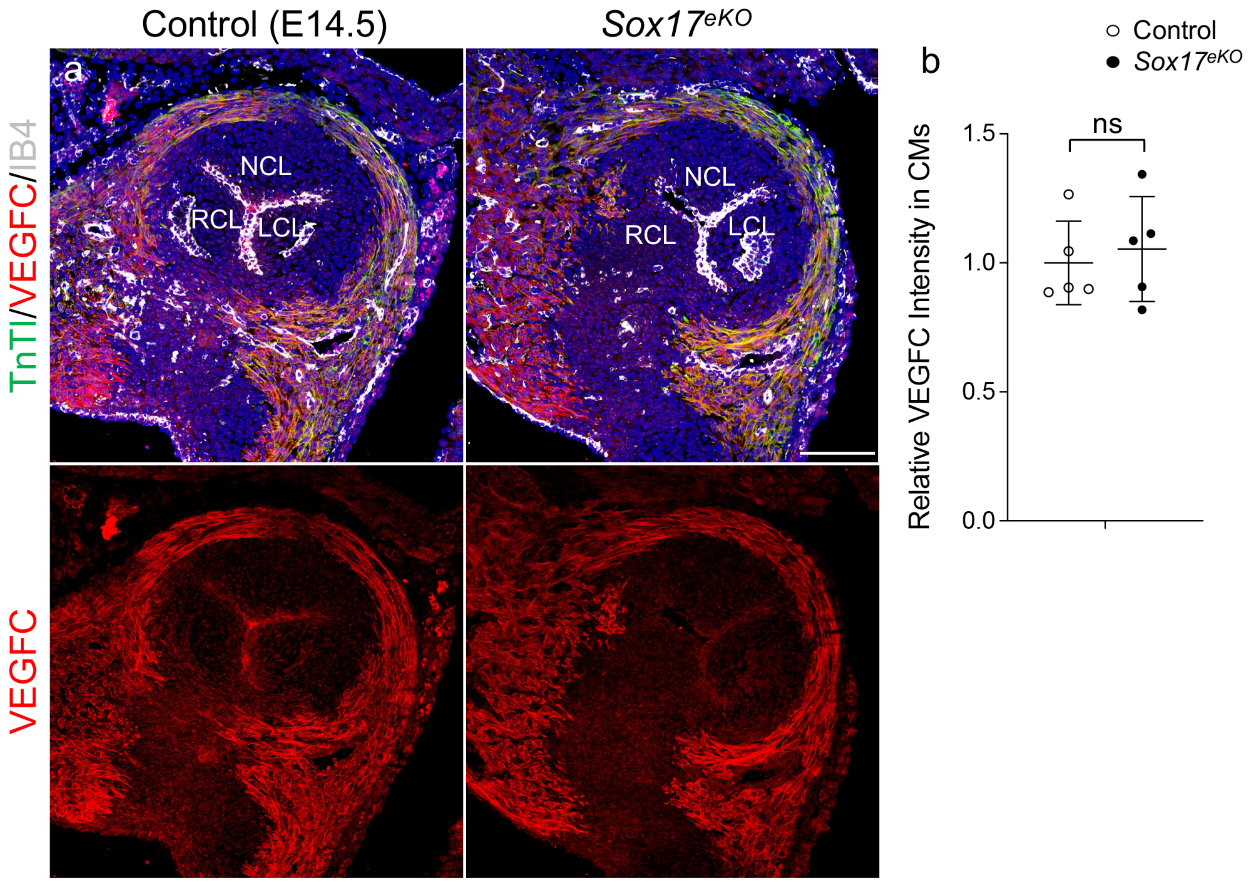

Supplementary Fig. 17. The *Sox17* deletion does not affect the expression of VEGFC. a, **b** Representative images and quantitative analysis of E14.5 control and *Sox17*^{eKO} hearts show comparable myocardial VEGFC (red) in the aortic region. TnTI (green) marks the myocardium. n=5/group, mean ± SD, unpaired two-tailed *t*-test, ns, no significance. Source data are provided as a Source Data file. Scale bars: 100 μm.

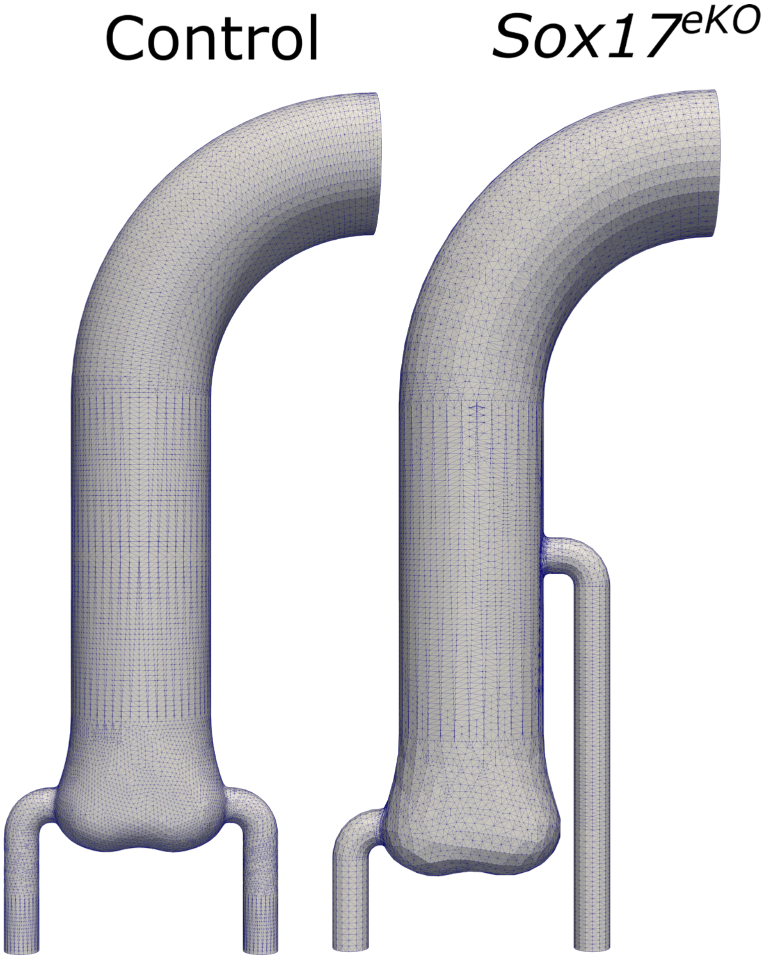

Supplementary Fig. 18. Idealized mesh geometries used in simulations. These models are constructed assuming diameters of 0.5 and 0.125 mm for the aorta and coronaries, respectively. The left mesh represents the effects of a normally placed LCO, whereas the right mesh represents the effects of the misplaced LCO present in the *Sox17*^{eKO} embryos.

Supplementary Tables

Supplementary Table 1. The survival summary of *Sox17^{eKO}* embryos

Stage	NO. litters	NO. embryos	KO (Died)
E12.5	7	59	10
E13.5	7	46	12
E14.5	24	168	43 (1)
E15.5	15	102	23 (1)
E16.5	23	157	33 (7)
E17.5	5	32	5 (3)
E18.5	4	26	6 (6)
P0-wean	21	111	0

The male *Nfatc1$^{enCre/+}$:Sox17$^{f/+}$* mice were crossed with the female *Sox17$^{f/f}$* mice. The percentage of *Sox17^{eKO}* embryos is expected at 25%, based on 1:4 Mendelian ratio from this cross.

Supplementary Table 2. List of antibodies and working conditions

Name	Manufacturer (Cat #)	Dilutions
SOX17	R&D (AF1924)	1:100
Isolectin-B4	Sigma (L-2140)	1:50
PECAM1	BD Pharmingen (550274)	1:100
TROPONIN I	Abcam (ab47003)	1:500
GFP	Abcam (ab6673)	1:500
ELASTIN	Abcam (ab21600)	1:100
Versican	Abcam (ab177480)	1:100
HABP2	Abcam (ab181837)	1:100
Collagen 1	Abcam (ab34710)	1:100
smMHC	BTI (BT-562)	1:300
KLF4	R&D (AF3158)	1:100
N1ICD	Cell signaling (#4147)	1:100
P-ERK1/2	Cell signaling (#9101)	1:100
ERK	Cell signaling (#9102)	1:100
PDGFRA	Abcam (ab203491)	1:100
PDGFRB	Abcam (ab32570)	1:100
ISL1	R&D (AF1837)	1:100
SOX7	R&D (AF2766)	1:100
VEGFC	R&D (AF752)	1:100

Supplementary Table 3. List of primers used for qPCR in this study

Gene	Primer sequence (5’ to 3’)	Size
Angptl4	CATCCTGGGACGAGATGAACT	136bp
	TGACAAGCGTTACCACAGGC	
Cnot3	GACAAGCGCAAACTCCAAGG	168bp
	CAGCCGTTGTAGCTTCTTAATCT	
Col15a1	CCCATTACCCTCGTCTGTGTC	120bp
	CTGAAGAAGGTCGGTGGGATG	
Cdk6	GGCGTACCCACAGAAACCATA	187bp
	AGGTAAGGGCCATCTGAAAACT	
Dcn	TAAAAGGTCGTGAAAATACAT	111bp
	GAAGTCAAATAAGCCTCTCTG	
Gja4	CCCACATCCGATACTGGGTG	220bp
	CGAAGACGACCGTCCTCTG	
Hhex	CTACACGCACGCCCTACTCC	104bp
	ACCTCACTTGACCGCCTTTCC	
Pkd2	GGGGAACAAGACTCATGGAAG	119bp
	GCCGTAGGTCAAGATGCACAA	
Pdgfra	TCCATGCTAGACTCAGAAGTCA	118bp
	TCCCGGTGGACACAATTTTTC	
Robo1	GCTGCCAAGCGGGTCTTTAT	101bp
	CTCCGAGGTAATTCCTAGCCA	
Sema3g	AGGTGGGGAGCTATACACAGG	128bp
	ACCGGGGTTCATGTAGGAGG	
Sox17	GGATGTAAAGGTGAAAGGCGA	197bp
	AAGACTTGCCTAGCATCTTGC	
Sox18	CCTGTCACCAACGTCTCGC	127bp
	GCAACTCGTCGGCAGTTTG	
Tnfaip3	GAACAGCGATCAGGCCAGG	105bp
	GGACAGTTGGGTGTCTCACATT	
Vcan	TTTTACCCGAGTTACCAGACTCA	106bp
	GGAGTAGTTGTTACATCCGTTGC	

Supplementary references

1. Aksoy I, *et al.* Oct4 switches partnering from Sox2 to Sox17 to reinterpret the enhancer code and specify endoderm. *The EMBO journal* **32**, 938-953 (2013).
