# Supplementary material for: A SOX17-PDGFB signaling axis regulates aortic root development
Source: Nat Commun. 2022 Jul 13;13:4065. doi: 10.1038/s41467-022-31815-1 (PMC9279414; doi:10.1038/s41467-022-31815-1)
Supplement: Supplementary file 2 — Reporting Summary [file 41467_2022_31815_MOESM2_ESM.pdf]

nature portfolio

Corresponding author(s): Bin Zhou
Last updated by author(s): Jun 3, 2022

Reporting Summary

Nature Portfolio wishes to improve the reproducibility of the work that we publish. This form provides structure for consistency and transparency in reporting. For further information on Nature Portfolio policies, see our Editorial Policies and the Editorial Policy Checklist.

Statistics

For all statistical analyses, confirm that the following items are present in the figure legend, table legend, main text, or Methods section.

n/a	Confirmed	
☐	☒	The exact sample size (n) for each experimental group/condition, given as a discrete number and unit of measurement
☐	☒	A statement on whether measurements were taken from distinct samples or whether the same sample was measured repeatedly
☐	☒	The statistical test(s) used AND whether they are one- or two-sided<br>*Only common tests should be described solely by name; describe more complex techniques in the Methods section.*
☒	☐	A description of all covariates tested
☒	☐	A description of any assumptions or corrections, such as tests of normality and adjustment for multiple comparisons
☐	☒	A full description of the statistical parameters including central tendency (e.g. means) or other basic estimates (e.g. regression coefficient) AND variation (e.g. standard deviation) or associated estimates of uncertainty (e.g. confidence intervals)
☐	☒	For null hypothesis testing, the test statistic (e.g. F, t, r) with confidence intervals, effect sizes, degrees of freedom and P value noted<br>*Give P values as exact values whenever suitable.*
☒	☐	For Bayesian analysis, information on the choice of priors and Markov chain Monte Carlo settings
☒	☐	For hierarchical and complex designs, identification of the appropriate level for tests and full reporting of outcomes
☐	☒	Estimates of effect sizes (e.g. Cohen's d, Pearson's r), indicating how they were calculated

Our web collection on statistics for biologists contains articles on many of the points above.

Software and code

Policy information about availability of computer code

Data collection	Fluorescence micrographs were taken on a Leica SP5 confocal microscope or Zeiss Observer Z1microscope.
Data analysis	Image J 1.48v, GraphPad Prism 8.3.0, AmiraTM software version 6.1.1

For manuscripts utilizing custom algorithms or software that are central to the research but not yet described in published literature, software must be made available to editors and reviewers. We strongly encourage code deposition in a community repository (e.g. GitHub). See the Nature Portfolio guidelines for submitting code & software for further information.

Data

Policy information about availability of data

All manuscripts must include a data availability statement. This statement should provide the following information, where applicable:
- Accession codes, unique identifiers, or web links for publicly available datasets
- A description of any restrictions on data availability
- For clinical datasets or third party data, please ensure that the statement adheres to our policy

The RNA-seq data has been deposited to the GEO database (GSE129564; https://www.ncbi.nlm.nih.gov/geo/query/acc.cgi?acc=GSE129564). SOX17 ChIP-seq reads in mouse embryonic stem cells overexpressing SOX17 were downloaded from the public GEO database (GSE43275; https://www.ncbi.nlm.nih.gov/geo/query/acc.cgi?acc=GSE43275). All other data supporting the findings of this study are available from the corresponding author on reasonable request.

Field-specific reporting

Please select the one below that is the best fit for your research. If you are not sure, read the appropriate sections before making your selection.

- [x] Life sciences
- [ ] Behavioural & social sciences
- [ ] Ecological, evolutionary & environmental sciences

For a reference copy of the document with all sections, see nature.com/documents/nr-reporting-summary-flat.pdf

Life sciences study design

All studies must disclose on these points even when the disclosure is negative.

Sample size	The sample sizes were chosen based on our experience with mouse aortic valve development in our previous published studies (Wang et al European Heart Journal, PMID: 26491108)
Data exclusions	No data exclusions. Both sexes of mice were used in the experiments.
Replication	All experiments were replicated in at least three times with pregnant females, at least 3-8 individual embryos were used fro each experiments. Specific numbers of replication done for each experiment are included in figures and figure legends.
Randomization	MCECs were randomly divided into SOX17 ChIP group and IgG ChIP group for the ChIP expriments.
Blinding	Blinding was applied to an independent researcher when analyzing the changes in phenotypes and gene expressions.

Reporting for specific materials, systems and methods

We require information from authors about some types of materials, experimental systems and methods used in many studies. Here, indicate whether each material, system or method listed is relevant to your study. If you are not sure if a list item applies to your research, read the appropriate section before selecting a response.

Materials & experimental systems

n/a	Involved in the study
[ ]	[x] Antibodies
[ ]	[x] Eukaryotic cell lines
[x]	[ ] Palaeontology and archaeology
[ ]	[x] Animals and other organisms
[x]	[ ] Human research participants
[x]	[ ] Clinical data
[x]	[ ] Dual use research of concern

Methods

n/a	Involved in the study
[x]	[ ] ChIP-seq
[x]	[ ] Flow cytometry
[x]	[ ] MRI-based neuroimaging

Antibodies

Antibodies used

Name Manufacturer (Cat #) Dilutions
SOX17 R&D (AF1924) 1:100
Isolectin-B4 Sigma (L-2140) 1:50
PECAM1 BD Pharmingen (550274) 1:100
TROPONIN I Abcam (ab47003) 1:500
GFP Abcam (ab6673) 1:500
ELASTIN Abcam (ab21600) 1:100
Versican Abcam (ab177480) 1:100
HABP2 Abcam (ab181837) 1:100
Collagen 1 Abcam (ab34710) 1:100
smMHC BTI (BT-562) 1:300
KLF4 R&D (AF3158) 1:100
N1ICD Cell signaling (#4147) 1:100
P-ERK1/2 Cell signaling (#9101) 1:100
ERK Cell signaling (#9102) 1:100
PDGFRA Abcam (ab203491) 1:100
PDGFRB Abcam (ab32570) 1:100
ISL1 R&D (AF1837) 1:100
SOX7 R&D (AF2766) 1:100
VEGFC R&D (AF752) 1:100
donkey anti-rat Alexa Fluor 488 (Thermo Fisher Scientific, A-21208, 1:200),

	donkey anti-rat Alexa Fluor 594 (Thermo Fisher Scientific, A-21209, 1:200),<br>donkey anti-rabbit Alexa Fluor 488 (Thermo Fisher Scientific, A-21206, 1:200),<br>donkey anti-rabbit Alexa Fluor 568 (Thermo Fisher Scientific, A-10042, 1:200),<br>donkey anti-goat Alexa Fluor 488 (Thermo Fisher Scientific, A-11055, 1:200),<br>donkey anti-goat Alexa Fluor 594 (Thermo Fisher Scientific, A-11058, 1:200),<br>Goat anti-mouse Alexa Fluor 488 (Thermo Fisher Scientific, A28175, 1:200)
Validation	All antibodies were purchased from the commercial vendors, validation information is available from manufactures' websites.<br>SOX17:https://www.rndsystems.com/products/human-sox17-antibody_af1924<br>Isolectin-B4:https://www.sigmaaldrich.com/US/en/product/sigma/l2140<br>PECAM1:https://www.bdbiosciences.com/en-us/products/reagents/flow-cytometry-reagents/research-reagents/single-color-antibodies-ruo/purified-rat-anti-mouse-cd31.550274<br>TROPONIN I:https://www.abcam.com/cardiac-troponin-i-antibody-ab47003.html<br>GFP:https://www.abcam.com/gfp-antibody-ab6673.html<br>ELASTIN:https://www.abcam.com/tropoelastin-antibody-ab21600.html<br>Versican:https://www.abcam.com/versican-antibody-epr12277-ab177480.html<br>HABP2:https://www.abcam.com/habp2-antibody-epr14551-ab181837.html<br>Collagen 1:https://www.abcam.com/collagen-i-antibody-ab34710.html<br>smMHC:https://fnkprddata.blob.core.windows.net/domestic/data/datasheet/BTI/BT-562.pdf<br>KLF4:https://www.rndsystems.com/products/mouse-klf4-antibody_af3158<br>N1ICD:https://www.cellsignal.com/products/primary-antibodies/cleaved-notch1-val1744-d3b8-rabbit-mab/4147<br>P-ERK1/2:https://www.cellsignal.com/products/primary-antibodies/phospho-p44-42-mapk-erk1-2-thr202-tyr204-antibody/9101<br>ERK:https://www.cellsignal.com/products/primary-antibodies/p44-42-mapk-erk1-2-antibody/9102<br>PDGFRA:https://www.abcam.com/pdgfr-alpha-antibody-epr22059-270-ab203491.html<br>PDGFRB:https://www.abcam.com/pdgfr-alpha--pdgfr-beta-antibody-y92-c-terminal-ab32570.html<br>ISL1:https://www.rndsystems.com/products/human-islet-1-antibody_af1837<br>SOX7:https://www.rndsystems.com/products/human-sox7-antibody_af2766<br>VEGFC:https://www.rndsystems.com/products/human-vegf-c-antibody_af752<br>donkey anti-rat Alexa Fluor 488:https://www.thermofisher.com/antibody/product/Donkey-anti-Rat-IgG-H-L-Highly-Cross-Adsorbed-Secondary-Antibody-Polyclonal/A-21208<br>donkey anti-rat Alexa Fluor 594:https://www.thermofisher.com/antibody/product/Donkey-anti-Rat-IgG-H-L-Highly-Cross-Adsorbed-Secondary-Antibody-Polyclonal/A-21209<br>donkey anti-rabbit Alexa Fluor 488:https://www.thermofisher.com/antibody/product/Donkey-anti-Rabbit-IgG-H-L-Highly-Cross-Adsorbed-Secondary-Antibody-Polyclonal/A-21206<br>donkey anti-rabbit Alexa Fluor 568:https://www.thermofisher.com/antibody/product/Donkey-anti-Rabbit-IgG-H-L-Highly-Cross-Adsorbed-Secondary-Antibody-Polyclonal/A10042<br>donkey anti-goat Alexa Fluor 488:https://www.thermofisher.com/antibody/product/Donkey-anti-Goat-IgG-H-L-Cross-Adsorbed-Secondary-Antibody-Polyclonal/A-11055<br>donkey anti-goat Alexa Fluor 594:https://www.thermofisher.com/antibody/product/Donkey-anti-Goat-IgG-H-L-Cross-Adsorbed-Secondary-Antibody-Polyclonal/A-11058<br>Goat anti-mouse Alexa Fluor 488:https://www.thermofisher.com/antibody/product/Goat-anti-Mouse-IgG-H-L-Secondary-Antibody-Recombinant-Polyclonal/A28175

Eukaryotic cell lines

Policy information about cell lines

Cell line source(s)	Immortalized Mouse Cardiac Endothelial Cells (MCEC) (CEDARLANE, Cat #: CLU510), prepared from microvascular neonatal mouse cardiac endothelial cells by transfection with lentiviral vectors carrying SV40 T antigen and human telomerase
Authentication	Immortalized Mouse Cardiac Endothelial Cells (MCEC) were purchased from CEDARLANE and were not authenticated.
Mycoplasma contamination	MCEC were negative for mycoplasma contamination.
Commonly misidentified lines<br>(See ICLAC register)	No commonly misidentified lines were used in this study.

Animals and other organisms

Policy information about studies involving animals; ARRIVE guidelines recommended for reporting animal research

Laboratory animals	The 3-6 month old both sex Nfatc1enCre, Sox17f/f, R26RfsGFP, TnTCre, R26RfshPDGFB mouse lines were used and maintained on a C57BL/6 background.
Wild animals	No wild animals were used in this study.
Field-collected samples	No field collected samples were used in this study.
Ethics oversight	Mouse holding and experimentation were performed according to protocols approved by the Institutional Animal Care and Use Committee (IACUC) of Albert Einstein College of Medicine.

Note that full information on the approval of the study protocol must also be provided in the manuscript.
