# Supplementary material for: A SOX17-PDGFB signaling axis regulates aortic root development
Source: Nat Commun. 2022 Jul 13;13:4065. doi: 10.1038/s41467-022-31815-1 (PMC9279414; doi:10.1038/s41467-022-31815-1)
Supplement: Supplementary file 3 — Description of Additional Supplementary Files [file 41467_2022_31815_MOESM3_ESM.pdf]

Description of Additional Supplementary Files

File Name: Supplementary Data 1
Description: RNA-seq related data including DEGs, Sox17 binding site, GO enrichment terms and genes
